# Scents on the slope: declining volatile emissions but stable predator-associated cues across elevation in alpine willows

**DOI:** 10.3389/fpls.2026.1873253

**Published:** 2026-07-29

**Authors:** Shristee Panthee, Priscila Mezzomo, Sofian A. Renoult, Monica Barman, Jing Vir Leong, Pavel Sebek, Petr Koutecký, Elvira Hörandl, Natascha D. Wagner, Petr Nguyen, Nicole M. van Dam, Martin Volf

**Affiliations:** 1 Plant-biotic Interactions, Leibniz Institute for Horticultural Sciences (IGZ), Großbeeren, Germany; 2 Institute of Biodiversity, Ecology and Evolution (IBEE), Friedrich Schiller University, Jena, Germany; 3 Institute of Entomology, Biology Centre Czech Academy of Sciences, Ceske Budejovice, Czechia; 4 Faculty of Science, University of South Bohemia, Ceske Budejovice, Czechia; 5 Department of Systematics, Biodiversity and Evolution of Plants (with Herbarium), University of Goettingen, Göttingen, Germany

**Keywords:** elevational gradient, induced indirect defenses, natural enemies, plasticine caterpillar, predation, *Salix*, volatile organic compounds

## Abstract

Volatile organic compounds (VOCs) can play important roles as indirect defenses and attract natural enemies of herbivores to induced plants. The relative importance of VOCs as indirect defense can be altered by abiotic conditions, plant identity, and the composition of local natural enemies. Elevational gradients provide an effective framework for investigating how plants allocate resources to VOC production and how these compounds influence the attraction of natural enemies of invertebrate herbivores under varying biotic and abiotic conditions. We studied induced VOCs and predation on four willow species along an elevational gradient in the Austrian Alps. We grouped the species into two pairs based on their ploidy (*Salix appendiculata-caprea* and *Salix mielichhoferi-myrsinifolia*), each pair collectively spanning the whole elevational gradient (1,000 to 2,230 m a.s.l.). We induced the plants with methyl jasmonate and quantified their induced VOC profiles. We measured vertebrate and invertebrate predation on yellow, green and brown colored dummy caterpillars, representing the diversity of caterpillars that typically feed on willows. Induced VOC emissions tended to decrease with elevation, with a clear decline in the *S. appendiculata–caprea* pair and a weaker, marginal tendency in the *S. mielichhoferi–myrsinifolia* pair. Overall predation rates on artificial caterpillars did not vary consistently with elevation. They were, however, influenced by caterpillar color and willow species identity, particularly in the *Salix mielichhoferi-myrsinifolia* pair, where yellow caterpillars experienced significantly lower predation rates at higher elevations. Predation rates correlated with both overall and individual VOC emissions. These relationships were partly explained by willow species and predation types, but largely independent of elevation. Emissions of individual VOCs associated with predation did not decrease with elevation. Overall, our results thus show that although total induced VOC emissions declined with elevation, predator-associated VOCs remained relatively stable and predicted predation independent of elevation. This suggests that willows may maintain key components of their induced VOC blends even where overall emissions are reduced. Our results indicate that VOC-mediated attraction of natural enemies can persist across heterogeneous mountain environments, supporting the role of induced VOCs as indirect defenses along elevational gradients.

## 1 Introduction

Plants produce diverse volatile organic compounds (VOCs) that mediate interactions with other organisms (Turlings and Erb, 2018). Many of these VOCs are induced by external stimuli, particularly insect herbivory, and form an important component of induced plant defenses (Unsicker et al., 2015; Mezzomo et al., 2024). Induced VOCs can directly affect insect herbivores by altering their feeding or oviposition preferences (Mozuraitis et al., 2002). Such VOCs can also function as indirect defense by signaling herbivore presence to natural enemies. Attraction to induced VOCs is well documented in invertebrate predators and parasitoids (Du et al., 1998; Turlings and Erb, 2018). Meanwhile, vertebrate predators such as birds may rely on both VOCs and visual cues when locating prey (Amo et al., 2013; Mäntylä et al., 2017). Beyond attracting natural enemies, induced VOCs can influence interactions among plants by priming or inducing defenses in neighbors, positioning them as key mediators of both direct and indirect plant defense in natural communities (Hagiwara et al., 2024). The ecological effectiveness of induced VOCs as infochemicals depends on abiotic conditions, plant identity, and the composition of local natural enemy assemblages (Pellissier et al., 2016; Douma et al., 2019). Therefore, the contribution of induced VOCs to plant defense likely varies along ecological gradients such as elevation. While some studies have examined how indirect plant defenses and associated trophic interactions change along environmental gradients, elevational patterns in induced VOC emissions and their attractiveness to natural enemies, particularly in woody plants, remain poorly understood (Pellissier et al., 2016; Kergunteuil et al., 2019). Thus, understanding elevational variation in induced VOC emissions and their relationship with predation is essential for explaining context dependence in plant defensive strategies, particularly in heterogenous habitats.

Elevational gradients provide a useful framework for studying how plant functional traits and chemical defenses respond to abiotic and biotic conditions (Defossez et al., 2018; Volf et al., 2022a). At higher elevations, plants experience shorter growing seasons, harsher climates, and limited resources. These conditions may favor greater investment in constitutive defenses with direct effects on herbivores (Pellissier et al., 2016; Volf et al., 2022a). The efficacy of induced VOCs may also decline at high elevations due to strong winds, low temperatures, elevated ozone, and high UV radiation, which affect VOC biosynthesis, inducibility, emission, and atmospheric stability (Pellissier et al., 2016; Douma et al., 2019). In contrast, plants at lower elevations benefit from greater resource availability for compensatory regrowth, which may favor greater investment in inducible VOCs (Pellissier et al., 2016). Beyond shifts in total VOC emissions, elevational clines may also occur in VOC richness and composition. Lowland environments often promote greater chemical divergence among plants, whereas stronger abiotic filtering at high elevations may constrain plants toward more similar chemical profiles (Pellissier et al., 2012; Volf et al., 2023). In addition, higher herbivore richness in lowland communities may promote greater variation in VOC blends among lowland plants, as the composition of induced VOCs depends on the identity of the attacking herbivore (Mezzomo et al., 2023). This specificity may allow plants to fine tune their defenses or selectively attract particular natural enemies (Mezzomo et al., 2026).

The importance of VOCs as indirect defense in high elevation plants may also be influenced by the generally lower abundance of natural enemies at higher elevations (Camacho and Avilés, 2019; Zvereva and Kozlov, 2022). Declines in predator abundance with elevation are particularly pronounced in some invertebrate predators, such as ants, due to decreasing temperatures and resources (Barlow et al., 2024; Camacho and Avilés, 2019). Higher wind speeds may further reduce the ability of flying predatory insects and parasitoids to locate their prey (Zvereva and Kozlov, 2022). In contrast, insectivorous birds occur across broader elevational ranges, with some species inhabiting very high altitudes. Although their abundance generally declines with elevation, species diversity often peaks at mid elevations (Klosius, 2008; Chamberlain et al., 2016; Sam et al., 2024). Additionally, predatory birds at higher elevations may show reduced prey selectivity or broader diets that include non-insect resources (Sam et al., 2024), potentially contributing to elevational changes in predation patterns through shifts in predator specialization.

Due to their wide elevational distribution, willows (*Salix* L.) represent an excellent system for studying elevational clines in VOC composition and their role in predator attraction. Willows often form diverse local assemblages of closely related species where co-occurrence is supported by their distinct chemical strategies (Leong et al., 2024). This mechanism appears particularly important in lowland species (Volf et al., 2023). Willows host diverse herbivore communities and produce diverse induced VOCs that vary depending on the identity of attacking herbivores (Inui et al., 2003; Mezzomo et al., 2023, 2024). Together with pronounced interspecific variation in induced VOC profiles, specificity in induced VOCs may contribute to the attraction of different predators to willows, including both invertebrates and insectivorous birds (Mrazova and Sam, 2018; Mezzomo et al., 2026). In addition to herbivory, willow constitutive VOC emissions can vary between low and high elevations, highlighting their sensitivity to abiotic conditions (Swanson et al., 2021).

Here, we explored elevational trends in induced VOCs and tested if they correlated with predation rates in four willow species along an elevational gradient spanning approximately 1,200 m in the Austrian Alps. We quantified elevational changes in induced VOC composition and differences among willow species. Predation was assessed using three differently colored artificial caterpillars representing the diversity of caterpillars that typically feed on willows. We first hypothesized that induced VOC emissions vary with willow species identity and elevation. Specifically, we expected VOC richness, total emissions, and composition to decline with elevation. Second, we hypothesized that predation rates decrease with elevation, particularly for invertebrate predators. As predators become less abundant and more opportunistic, we expected greater variation in overall predation but weaker differences among caterpillar colors toward higher elevations. Finally, we hypothesized that changes in predation would be explained by changes in VOCs. By testing these hypotheses, we aim to identify elevational patterns in VOC composition and evaluate how VOCs help willows cope with herbivory by attracting predators in different environments.

## 2 Materials and methods

### 2.1 Sampling sites and focal species

The study was conducted between July and August 2023 along an elevational transect covering a gradient from 1,000 to 2,230 m a.s.l. in the Möll Valley, Carinthia, Austria (**Supplementary Table 1**). We established 12 study sites, each approximately 0.25 ha, across the elevational gradient. These sites covered habitats from the montane to alpine zones, with the tree line located at approximately 1,900 to 2,000 m a.s.l (Körner, 2018; Körner, 2021). We focused on two pairs of closely related willow species that were particularly common along the gradient: (i) the diploid species *Salix appendiculata* Vill. and *S. caprea* L., and (ii) the hexaploid species *S. mielichhoferi* Saut. and *S. myrsinifolia* Salisb. Within each pair, the species collectively spanned the entire elevational gradient. Members of each pair are closely related and frequently hybridize or form morphologically intermediate individuals (Hörandl et al., 2012). At each site, we mapped and identified focal plants based on leaf, twig, and flower morphology following Hörandl et al. (2012). If present and accessible, we sampled up to five individuals per species, their hybrids or intermediate forms per site, resulting in 102 sampled plants. Selected plants were at least 5 m apart. We collected vouchers from all plants, which were later examined by three specialists (Elvira Hörandl, Petr Koutecký and Natascha D. Wagner) to improve field identifications. We further refined identifications by analyzing repetitive sequences with species-specific abundances using RepeatExplorer2 comparative repeat analysis of low coverage genomic data (**Supplementary Figures 1**, **2**) (Novák et al., 2020). See **Supplementary Material 1** for details. Molecular based identifications were used in all the subsequent analyses in this study.

### 2.2 Sampling of VOCs

We collected induced VOCs using passive headspace sampling following Kallenbach et al. (2014) (**Supplementary Figure 3**) from 102 willow individuals in total (**Supplementary Table 2**). For each plant, we selected an undamaged 30–45 cm branch with approximately 30 leaves. Before sampling VOCs, plants were once sprayed with an aqueous solution containing 5 mM methyl jasmonate till run off (MeJA; Sigma-Aldrich Chemie GmbH), 1% EtOH and 0.1% Triton X-100 (Sigma-Aldrich, St. Louis, Missouri, USA) (Volf et al., 2022b). MeJA is a phytohormone widely used to simulate herbivore-induced plant responses in ecological studies (Mrazova and Sam, 2018; Volf et al., 2022b). Exogenous application of MeJA has been shown to induce rapid quantitative and qualitative changes in VOC emissions, often producing blends that overlap with, but are not identical to, herbivore−induced bouquets in various woody plants including willows (Volf et al., 2021; Mrazova and Sam, 2018; Amo et al., 2022).

Immediately after evaporation of the treatment solution (approx. 4–5 minutes), two PDMS (polydimethylsiloxane) tubes (2 cm, outer diameter 1.8 mm; Carl Roth, Karlsruhe, Germany) were attached to each branch on a stainless-steel wire to prevent contact with plant surfaces. The tubes were enclosed in 25 × 38 cm polyamide bags (Alufix Bohemia, Cerniky, Czech Republic). VOCs were passively sampled from the headspace for 24 h, typically starting between 09:00 and 10:00 h. At each site, we also collected a non-leaf control sample from a dry, leafless branch. The branch was sprayed with MeJA, and VOCs were collected following the same procedure described above.

VOCs were analyzed using a 7890B gas chromatograph coupled to a 7010 Triple Quadrupole mass spectrometer (Agilent, Santa Clara, California, USA). GC-MS analysis followed Volf et al. (2021) with minor modifications. Compounds were thermally desorbed using a MultiPurpose Thermal Desorption sampler (Gerstel, Mülheim an der Ruhr, Germany) with a temperature program from 25 °C to 220 °C (60 °C min^-^¹, 8 min hold). Compounds were cryo-cooled in a cooled injection system (CIS, Gerstel) at −50 °C and transferred to the GC by heating to 230 °C (12 °C min^-^¹, 3 min hold). Separation was performed on an RTX Wax column (30 m × 0.25 mm, 0.25 µm film thickness; Restek, Bellefonte, Pennsylvania, USA) with helium carrier gas at 1 ml min^-^¹. The GC oven program was 60 °C (2 min), 30 °C min^-^¹ to 150 °C, 10 °C min^-^¹ to 200 °C, and 30 °C min^-^¹ to 230 °C (5 min hold). The MS transfer line was set to 240 °C and the ion source to 230 °C. Mass spectra were acquired in EI mode (70 eV) with a scan range of m/z 35–350.

Chromatograms were processed in Agilent MassHunter Qualitative Analysis (v10.0). Compounds were putatively identified using NIST 17, NIST 20 and Wiley 11 libraries and confirmed with in house standards and retention indices calculated from C8–C20 and C10–C40 alkane standards. VOCs detected in leaf samples were compared with non-leaf controls collected at the experimental sites and instrumental blanks (blank samples added in between leaf samples while processing in GC-MS-TD to improve compound detection). We then constructed a custom *Salix* VOC library containing mass spectra and retention indices for semi-automated detection and integration across samples. In house standards included GLVs ((*Z*)-3-hexen-1-ol and 3-hexen-1-ol acetate), monoterpenes (α- and β-pinene, β-myrcene, eucalyptol, limonene, linalool, (*E*)- and (*Z*)-β-ocimene, and γ-terpinene), aldehyde (nonanal), sesquiterpenes ((*Z*,*Z*)- and (*E*,*E*)-farnesene, β-caryophyllene and humulene) and the alkane (tetradecane). Before statistical analyses, peak areas were standardized by the total leaf area (cm²) enclosed in sampling bags.

### 2.3 Predation experiment

Following VOC sampling and bag removal, we initiated the predation experiment on the same trees. We selected three healthy branches per tree, excluding the branch used for collecting VOCs. Each branch received a pair of plasticine caterpillars (Büroshop, Nuremberg, Germany) in one of three colors: green, brown, or yellow with a brown ventral side (**Supplementary Figure 3**), to test for potential elevational shifts in predator preferences. These colors reflect common willow feeding caterpillars such as various *Orthosia* and *Operophtera* species for green, *Biston* and *Peribatodes* species for brown and *Erannis* and *Phalera* species for yellow. Caterpillars (3 cm long, 3 mm diameter) were shaped using a clay extruder and attached to branches with Loctite 401 super glue (Henkel, Düsseldorf, Germany). We then applied the MeJA treatment to the leaves of all selected branches where artificial caterpillars were placed to induce VOCs.

Predation marks were assessed twice at 5-day intervals, with MeJA treatment renewed during each visit. During the first visit, caterpillars were checked and repaired or replaced if missing or heavily damaged. At both visits, we recorded beak and bite marks and classified them as bird or invertebrate predation following Sam et al. (2015). Although we initially aimed to distinguish ants from other invertebrates, no marks attributable to ants were detected, so all invertebrate predation was combined into one category. Marks caused by surrounding vegetation or by non-predatory invertebrates (e.g., snails) were excluded.

### 2.4 Statistical analysis

As an exploratory step, we first used the VennDiagram package in R version 4.4.1 to descriptively visualize unique and shared VOCs among the four willow species (Chen and Boutros, 2011; R Core Team, 2025). To test our first hypothesis, which predicted differences in VOC composition among species and along the elevational gradient, we visualized overall variation in VOC profiles using Principal Component Analysis (PCA) based on standardized peak areas of detected compounds across samples. Because the PCA revealed pronounced differences between the two species pairs, all subsequent analyses were conducted separately for each pair. We then performed Redundancy Analysis (RDA), with species and elevation included as explanatory variables, log-transformed VOC emissions as response variables, and site as a covariate to account for variation among plant individuals associated with local site conditions. The significance of explanatory variables was tested using Monte Carlo permutation tests with 9,999 permutations. Variation explained by species and elevation was then partitioned using the vegan package in R (Oksanen et al., 2025).

To further test our first hypothesis, we examined whether VOC richness, total VOC emissions, and their variability changed along the elevational gradient. We first tested for elevational changes in VOC richness and total VOC emissions using Generalised Linear Mixed Models (GLMMs) with a Gaussian distribution in the glmmTMB package (Brooks et al., 2017). VOC emissions were log-transformed before analysis. We used species identity, elevation, and their interaction as fixed effects, and site as a random effect. We compared this full model with reduced models including only species identity, only elevation, or only the random effect of site using likelihood ratio tests and AIC. We then tested whether variability in VOC richness and total emissions changed across elevations using Permutational Multivariate Analysis of Dispersion (PERMDISP) in the vegan package (Oksanen et al., 2025). This analysis allowed us to test whether samples became more or less variable in VOC richness and total emissions across the elevational gradient. To reduce zero inflation, VOCs occurring in fewer than 20% of samples were removed before analysis. Bray-Curtis dissimilarities were then calculated from the transformed data. Elevation was treated as a factor, with levels corresponding to the sampled elevation values from 1,000 to 2,230 m, to test for differences in multivariate dispersion among elevations. Significance was evaluated using 999 permutations. To further examine variability in VOCs along the gradient, we fitted Generalised Additive Models (GAMs) using the mgcv package (Wood, 2017). In these models, distances of samples to their group centroid, obtained from PERMDISP, were log-transformed and modelled with elevation as a continuous predictor. We used a conservative basis dimension, k = 3, to detect potential nonlinear relationships.

To test our second hypothesis, which predicted elevational changes in predation rates, we calculated predation rates at the tree level. Predation rates were calculated by dividing the number of predation events caused by birds or invertebrates by the total number of caterpillars checked per tree. This corresponded to a maximum of 12 caterpillars per tree across the two assessment periods (see **Supplementary Table 2**). Each tree had three branches, with two caterpillars placed on each branch during each assessment. Branch- and assessment-level observations were therefore aggregated to obtain a single predation rate per tree. We first examined overall trends in predation patterns using Redundancy Analysis (RDA) in the vegan package in R (Oksanen et al., 2025). Species identity and elevation were included as explanatory variables. Response variables were predation rates separated by predator type (bird or invertebrate), and caterpillar color (green, brown or yellow). Site was included as a covariate. The significance of explanatory variables was tested using Monte Carlo permutation tests with 9,999 permutations. We then fitted GLMMs to test the effects of elevation, predator type, caterpillar color, and willow species identity on predation rates, with site included as a random effect. Models were fitted with a binomial distribution using the glmmTMB package. Candidate models were compared with a null model including only the random effect of site using AIC. Pairwise *post-hoc* comparisons for significant factors were conducted using the emmeans package (Lenth, 2024).

As a complementary analysis under the second hypothesis, we examined whether elevational trends in predation differed among brown, green, and yellow caterpillars. We fitted generalized linear models (GLMs) with a binomial error distribution and logit link. Predation was modelled as a binary response for each caterpillar color at the tree level, indicating whether predation was detected or not, with caterpillar color, elevation, and their interaction included as fixed effects. Model fit and explanatory power were evaluated using Nagelkerke’s pseudo-R², calculated with the nagelkerke function in the rcompanion package (Mangiafico, 2025). We then tested whether variability in predation patterns changed with elevation using PERMDISP. Elevation was treated as a factor, with levels corresponding to the sampled elevation values from 1,000 to 2,230 m, to test for differences in multivariate dispersion among elevations. To further examine changes in variability along the gradient, we fitted GAMs using the mgcv package (Wood, 2017), with elevation modelled as a continuous predictor.

To test our third hypothesis, which predicted relationships between predation rates and VOC profiles, we first calculated distance matrices describing pairwise dissimilarities among plants in bird predation, invertebrate predation, VOC richness, total VOC emissions, and elevation. All matrices were calculated using the Bray-Curtis dissimilarity index. We then used partial Mantel tests to assess correlations between predation dissimilarity matrices, separately for birds and invertebrates, and dissimilarity matrices based on total VOC emissions or VOC richness, while controlling for elevational distance. Significance was assessed using Pearson correlations with 999 permutations in the vegan package (Oksanen et al., 2025). We further examined relationships between predation rates and individual VOCs. This analysis included only VOCs that occurred in more than 20% of samples and showed significant log_2_ fold differences between plant samples and non-leaf controls based on Welch’s t-tests. Bird and invertebrate predation rates were modelled using GLMMs with a beta-family distribution, with site included as a random factor and emissions of selected VOCs included as fixed predictors. Each model was compared with a corresponding null model using likelihood ratio tests and AIC. To reduce the risk of false positives from multiple testing, we first retained only models in which inclusion of a VOC improved model fit by more than 2 AIC units. We then applied false discovery rate (FDR) correction to the remaining tests. For VOCs that showed significant relationships with predation rates, we further analyzed their elevational trends using GAMs, following the same approach as for total VOC emissions. All analyses were performed in R version 4.5.1.

## 3 Results

### 3.1 Volatile organic compounds

We identified 42 VOCs across the studied willow species (**Supplementary Table 3**). Of these, 40 occurred within the *Salix appendiculata–caprea* pair and 39 within the *S. mielichhoferi–S. myrsinifolia* pair (**Supplementary Figure 4**). At the species level, *Salix caprea* showed the highest VOC richness (40 compounds), followed by *S. mielichhoferi* (39), *Salix appendiculata* (35), and *S. myrsinifolia* (34). Most induced VOCs were sesquiterpenes, followed by monoterpenes, green leaf volatiles (GLVs), benzenoids, alkanes and aldehydes (**Supplementary Table 3**).

**Figure 1:**
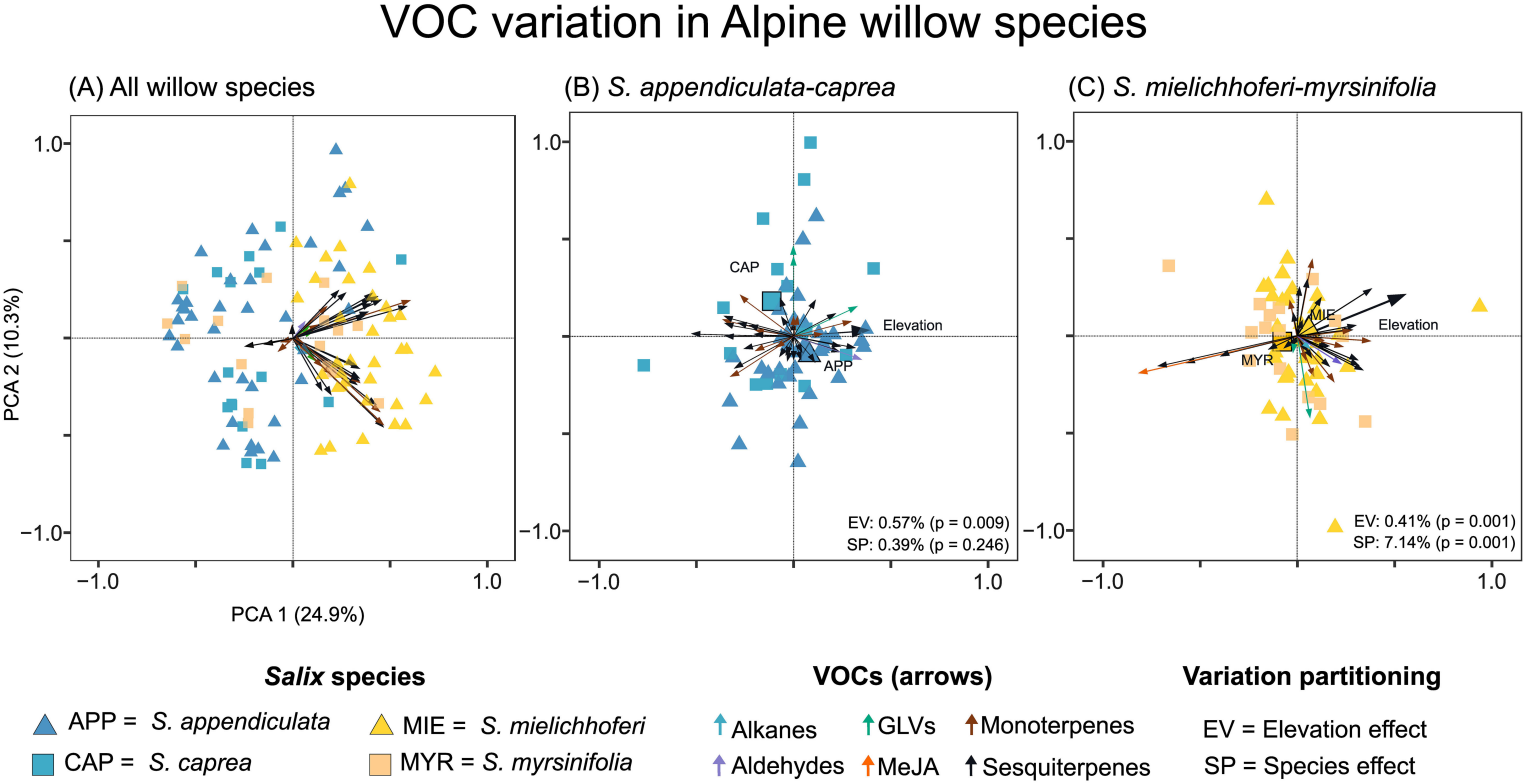
**(A)** Variation in VOC emissions across four willow species (*Salix appendiculata*, *S. caprea*, *S. mielichhoferi*, *S. myrsinifolia*). The first two PCA axes explained 35.2% of the variation. **(B)** RDA of the *S. appendiculata–caprea* pair showing a significant effect of elevation (0.59%, pseudo-F = 2.21, p = 0.009). **(C)** RDA of the *S. mielichhoferi–myrsinifolia* pair showing a significant effect of elevation (0.41%, pseudo-F = 2.87, p = 0.001) and species identity (7.14%, pseudo-F = 4.85, p = 0.001). Different shapes and colors are used for species pair and species differentiation. The colored arrows represent different VOC classes.

Across all four *Salix* species, the first two PCA axes explained 35.2% of the total variation in VOC profiles (**Figure 1A**). Because VOC profiles differed significantly between the two species pairs, we analyzed them separately in subsequent analyses. In the *S. appendiculata-caprea* pair, RDA showed that elevation explained 0.59% of variation in VOC composition and had a significant effect (pseudo-F = 2.21, p = 0.009), whereas species identity was not significant (pseudo-F = 1.19, p = 0.246; **Figure 1B**). In contrast, in the *S. mielichhoferi-myrsinifolia* pair, both elevation and species identity significantly explained variation in VOC composition. Elevation explained 0.41% of the variation (pseudo-F = 2.87, p = 0.001), whereas species identity explained 7.14% (pseudo-F = 4.85, p = 0.001; **Figure 1C**).

**Figure 2:**
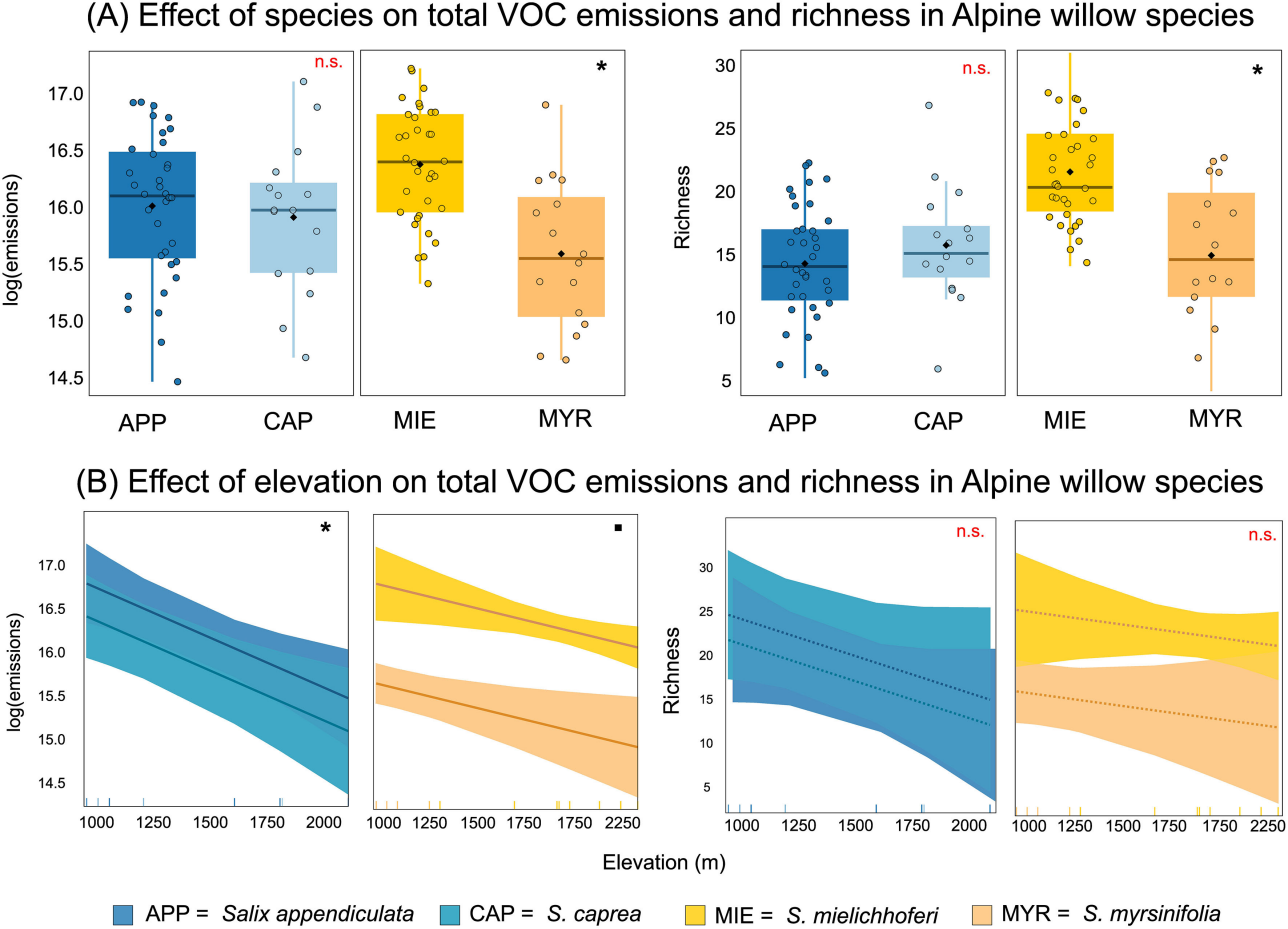
**(A)** Species differences in VOC emissions and richness within the *S. appendiculata–caprea* and *S. mielichhoferi–myrsinifolia* pairs. In the latter pair, species identity significantly affected VOC emissions ((χ²(4) = 24.49, p < 0.001) and richness (χ²(4) = 7.058, p = 0.008) in GLMMs. **(B)** Elevational trends in VOC emissions and richness. VOC emissions decreased significantly with elevation in *S. appendiculata–caprea* pair (χ²(3) = 10.57, p = 0.001) and showed marginal tendency to decrease with elevation in *S. mielichhoferi–myrsinifolia* pair (χ²(3) = 3.73, p = 0.053). Significant results are marked with asterisks (*), marginal results with points (.), and non-significant results are with “n.s.” (non-significant) on the top right corner and dashed trend lines.

In the *S. appendiculata–caprea* pair, total VOC emissions were significantly explained by the full GLMM model including species and elevation (χ²(4) = 14.17, p = 0.0008). However, species had no independent effect based on the model excluding the effect of elevation (χ²(3) = 0.28, p = 0.595; **Figure 2A**), whereas emissions decreased with elevation (χ²(3) = 10.57, p = 0.001; **Figure 2B**). VOC richness was neither explained by the full model (χ²(5) = 4.31, p = 0.115) nor by species (χ²(4) = 2.23, p = 0.135; 2A) and elevation (χ²(4) = 3.04, p = 0.08; 2B). In the *S. mielichhoferi–myrsinifolia* pair, total VOC emissions were explained by the full model (χ²(4) = 24.49, p < 0.001). Species had significant effect on emissions (χ²(3) = 18.49, p < 0.001; **Figure 2A**) and it showed a weak, marginal tendency to decrease with elevation (χ²(3) = 3.73, p = 0.053; **Figure 2B**). Richness was not explained by full model (χ²(5) = 7.74, p = 0.021), however, it was better explained by independent species model (χ²(4) = 7.058, p = 0.008; **Figure 2A**) and showed no elevational change (χ²(4) = 1.87, p = 0.174; **Figure 2B**).

VOC variability differed among elevations in the *S. appendiculata–caprea* pair (PERMDISP; F(7,44) = 4.132, p = 0.003; **Supplementary Figure 5**), with GAM trend indicating a significant non-linear elevational decrease in VOC variability (χ²(1.99) = 0.046, p = 0.001; **Supplementary Figure 5**). No elevational changes in VOC variability were detected in the *S. mielichhoferi–myrsinifolia* pair (PERMDISP; F(11,38) = 1.678, p = 0.128; **Supplementary Figure 5**).

### 3.2 Predation trends

**Figure 3:**
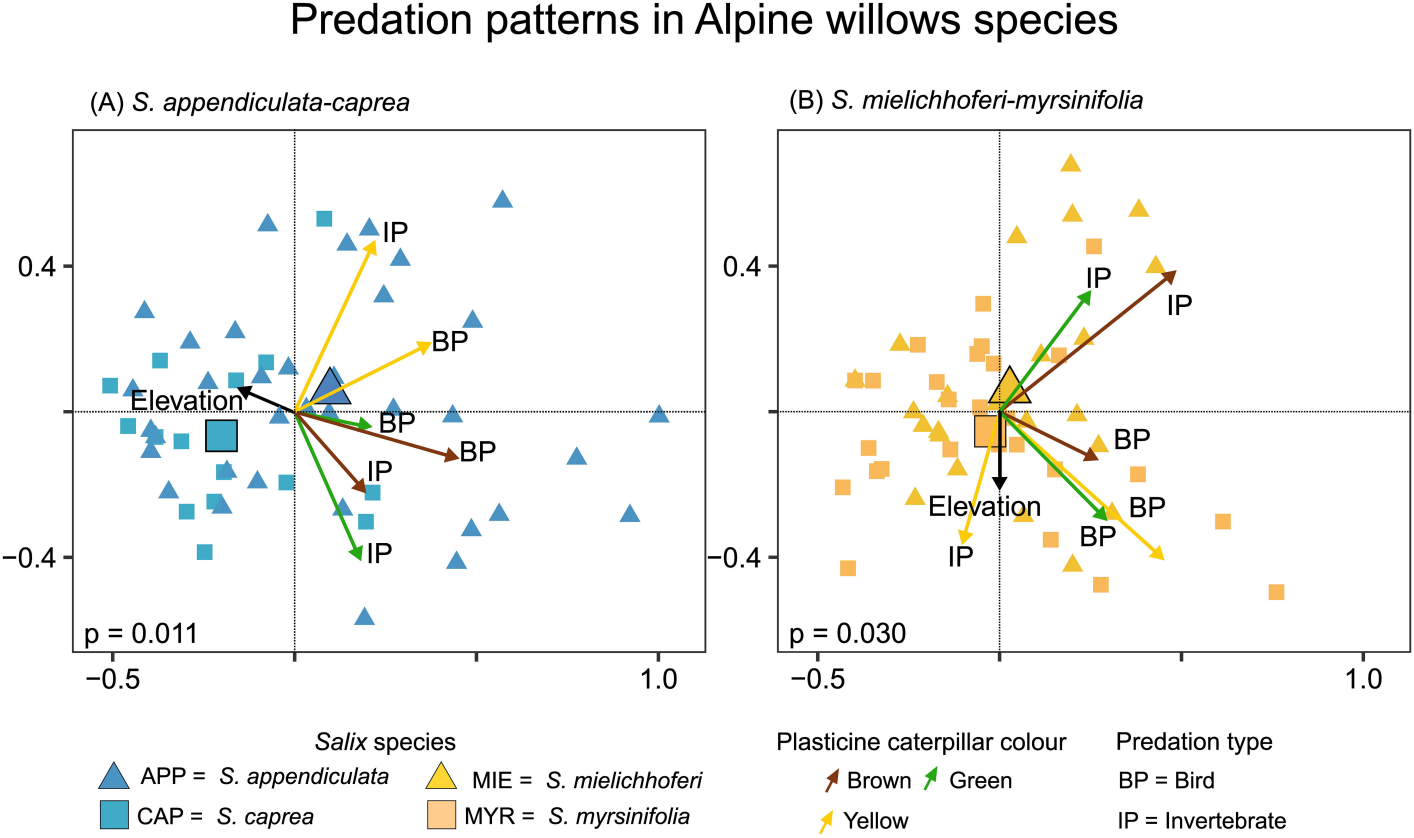
Variation in bird and invertebrate predation on green, brown, and yellow plasticine caterpillars as explained by elevation and willow species (RDA). **(A)** In the *S. appendiculata–caprea* pair, elevation and species explained 5.09% of variation (pseudo-F = 2.29, p = 0.011). **(B)** In the *S. mielichhoferi–myrsinifolia* pair, the same variables explained 4.11% of variation (pseudo-F = 1.91, p = 0.0304). Different shapes and colors are used for species pair and species differentiation. The colored arrows represent different caterpillar colors.

**Figure 4:**
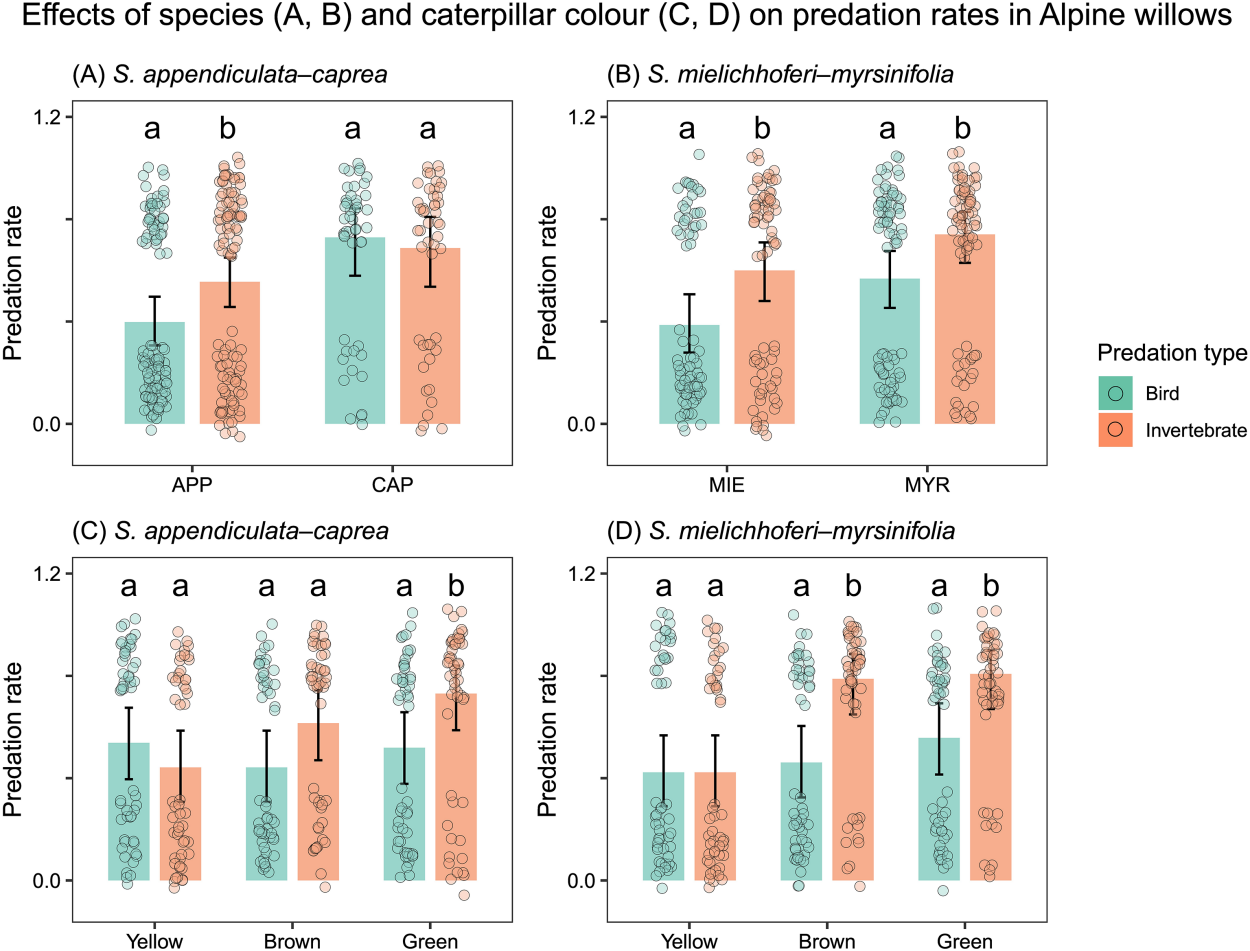
Effects of willow species identity and caterpillar color on bird and invertebrate predation in the *S. appendiculata–caprea* and *S. mielichhoferi–myrsinifolia* pairs (GLMMs). **(A, B)** Willow species identity significantly affected predation in both pairs (A: χ²(3) = 13.035, p = 0.005; B: χ²(5) = 13.58, p = 0.003). **(C, D)** Caterpillar color also had a significant effect on predation in both pairs, with a weaker effect in *S. appendiculata–S. caprea* (C: χ²(7) = 14.23, p = 0.0141) and a stronger effect in *S. mielichhoferi–S. myrsinifolia* (D: χ²(7) = 39.48, p < 0.001). Significant differences are indicated by different letters above box plots within each panel, with colors denoting bird (green) and invertebrate (orange) predation.

Across all four willow species and elevations, the mean overall predation rate was 0.47 (± 0.26) per tree. When divided by colors it was 0.58 (± 0.40) on green, 0.49 (± 0.36) on brown, and 0.35 (± 0.34) on yellow caterpillars. Birds accounted for about 43.65% of attacks and invertebrates for 56.35%. When analyzing predation types and caterpillar colors separately, elevation and willow species together explained 5.09% of the variation in the *S. appendiculata–caprea* pair (RDA; pseudo-F = 2.29, p = 0.011; **Figure 3A**) and 4.11% in the *S. mielichhoferi–myrsinifolia* pair (pseudo-F = 1.91, p = 0.030; **Figure 3B**). GLMMs showed that plant species identity was the best predictor of overall predation in the *S. appendiculata–caprea* pair (χ² (3) = 13.035, p = 0.005; **Figure 4A**). However, *post-hoc* tests indicated that this effect resulted from differences among predation types within species, as overall predation did not differ between the two willow species. Caterpillar color also affected predation (χ² (7) = 14.23, p = 0.0141; **Figure 4C**). In the *S. mielichhoferi–myrsinifolia* pair, caterpillar color was the best predictor of overall predation (χ² (7) = 39.48, p < 0.001; **Figure 4D**). *Post-hoc* tests showed that invertebrate predation was higher on brown and green caterpillars than on yellow ones, whereas bird predation did not differ among colors (**Supplementary Table 4**). Plant species identity also had a significant effect (χ² (5) = 13.58, p = 0.003; **Figure 4B**), but this model had higher AIC than the color model, and species differences were driven by interactions with predation type rather than by overall differences between willow species.

In the *S. appendiculata–caprea* pair, elevational trends in overall predation did not differ between yellow and green caterpillars (SE = 1.06, z = 1.70, p = 0.089), yellow and brown caterpillars (SE = 1.04, z = 0.95, p = 0.34) or green and brown caterpillars (SE = 0.28, z = –1.41, p = 0.34). In the *S. mielichhoferi–myrsinifolia* species pair, elevational trends in overall predation on yellow caterpillars did not differ from those on green (SE = 1.07, z = –0.14, p = 0.89) and brown caterpillars (SE = 1.04, z = –0.65, p = 0.515), however, yellow caterpillars showed lower predation at higher elevations (Nagelkerke R² = 0.82, LR χ²(5) = 451.0, p < 0.001; **Supplementary Figure 6**). Elevational trends did not differ between green and brown caterpillars (SE = 0.29, z = –0.88, p = 0.65).

**Figure 5:**
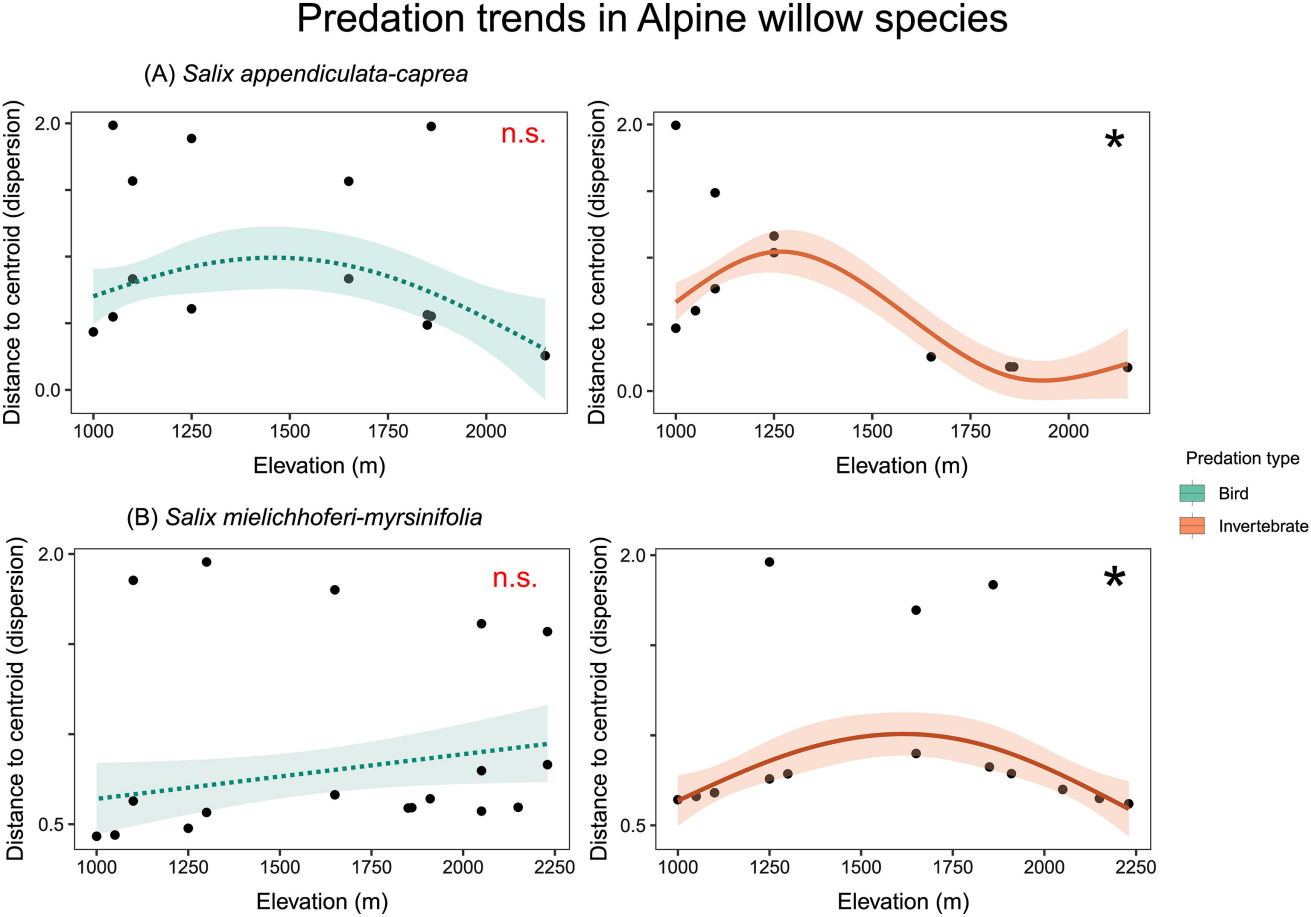
Elevational trends in variability in bird and invertebrate predation. **(A)** In the *S. appendiculata–caprea* pair, bird predation variability peaked at mid elevations (GAM: adj. R² = 0.12, F = 3.12, p = 0.044), whereas invertebrate predation variability decreased with elevation (PERMDISP; F(2) = 4.38, p = 0.014, GAM: adj. R² = 0.59, F = 25.4, p < 0.001). **(B)** In the *S. mielichhoferi–myrsinifolia* pair, bird predation variability didn’t change (PERMDISP: F(2) = 1.37, p = 0.23), while invertebrate predation variability peaked at mid elevation and decreased at higher elevation (dispersion GAM: adj. R² = 0.26, F = 6.68, p = 0.001). Significant results are marked with asterisks (*) and non-significant results are with “n.s.” (non-significant) on the top right corner and dashed trend lines.

In the *S. appendiculata–caprea* pair, PERMDISP did not detect elevational changes in bird predation variability (F(2) = 0.43, p = 0.66). GAM analysis nevertheless showed that bird predation variability peaked at mid elevations (dispersion GAM: adj. R² = 0.12, F = 3.12, p = 0.044; **Figure 5A**). In contrast, invertebrate predation variability did not change with elevation (PERMDISP, F(2) = 4.38, p = 0.014), showing an elevational decrease in variability (dispersion GAM: adj. R² = 0.59, F = 25.4, p < 0.001; **Figure 5A**). In the *S*. *mielichhoferi*–*myrsinifolia* pair, we did not detect elevational changes in bird predation variability (PERMDISP: F(2) = 0.62, p = 0.53, dispersion GAM: adj. R² = 0.041, F = 3.23, p = 0.078; **Figure 5B**). Invertebrate predation variability did not changed with elevation (PERMDISP: F(2) = 1.37, p = 0.23), however, Gam analysis showed higher variability at mid elevations and lower variability at the highest elevation (dispersion GAM: adj. R² = 0.26, F = 6.68, p = 0.001; **Figure 5B**).

### 3.3 Correlation between VOCs and predation

In the *S. appendiculata–caprea* pair, invertebrate predation positively correlated with total VOC emissions (r = 0.098, p = 0.035) in partial Mantel tests accounting for elevation (**Supplementary Table 5**). In the *S. mielichhoferi–myrsinifolia* pair, invertebrate predation correlated with total VOC richness (r = 0.137, p = 0.024). Similarly, invertebrate predation on brown caterpillar positively correlated with VOC richness (r = 0.21, p = 0.01) and on green caterpillar positively correlated with VOC emissions (r = 0.089, p = 0.048). Other correlations were not significant. When testing individual VOCs, β-bourbonene correlated with bird predation in the *S*. *appendiculata*–*caprea* pair (ΔAIC = 5.81, χ² = 7.81, p = 0.0078) and (Z)-3-hexen-1-ol acetate (ΔAIC = 5.89, χ² = 7.89, p = 0.0078) and α-muurolene correlated with bird predation in the *S*. *mielichhoferi*–*myrsinifolia* pair (ΔAIC = 2.56, χ² = 4.56, p = 0.033). No individual VOCs showed significant correlations with invertebrate predation in either species pair (**Supplementary Table 6**). None of the individual VOCs correlated to predation showed significant elevational trends (**Supplementary Table 7**).

## 4 Discussion

The roles of plant VOCs in recruiting natural enemies are well established in controlled settings such as laboratories and greenhouses (Turlings and Erb, 2018). However, their ecological functions along natural elevational gradients remain relatively poorly understood. We therefore investigated induced VOC emissions and predation patterns in two pairs of willow species along an elevational gradient. Our results indicate that elevation shapes induced VOC profiles, but that predation responses are mediated by willow species identity, predator type, and prey color rather than elevation alone. Importantly, although total VOC emissions significantly or marginally declined with elevation, individual induced VOCs correlated to predation did not.

Previous studies have reported variable elevational patterns in plant VOC emissions (Pellissier et al., 2016; Galmán et al., 2021). Constitutive VOC emissions may increase with elevation, show no consistent elevational trend, or differ in the emission rates of particular compounds or compound groups (Galmán et al., 2021; Simin et al., 2022; Rieksta et al., 2023). For example, Rieksta et al. (2023) found that dwarf birch at higher elevations emitted less diverse VOC blends but showed similar or higher emission rates than plants at lower elevations. These findings suggest that high-elevation conditions do not necessarily reduce VOC production in a simple way. Instead, elevational responses may involve partly independent changes in emission intensity, VOC richness, and blend composition. Our results are consistent with this view. In willows induced with methyl jasmonate, total VOC emissions declined significantly with elevation in the *S. appendiculata-caprea* pair and showed a weak marginal decline in the *S. mielichhoferi-myrsinifolia* pair. In the *S. mielichhoferi-myrsinifolia* pair, elevation was also associated with changes in VOC composition. However, VOC richness did not show a consistent elevational decrease. Thus, elevational responses in induced willow VOCs were expressed mainly through changes in emission intensity and, in one species pair, blend composition, rather than through a clear reduction in the number of detected compounds. The contrast between our results on induced emissions and previous studies on constitutive emissions may indicate that willows at higher elevations rely less on inducible VOC responses, similarly to trends observed in other plants (Pellissier et al., 2016; Defossez et al., 2018). Testing this interpretation would, however, require direct measurements of constitutive VOC emissions, which were not included in our study because our primary aim was to examine relationships between induced VOC profiles and predation.

Beyond changes in total emissions and VOC composition, we also detected changes in overall VOC variation along the elevational gradient. In the *S. appendiculata-caprea* pair, this variation followed a non-linear elevational trend, peaking at mid elevations and gradually decreasing toward higher elevations. This pattern resembles the elevational decrease in variation reported for several non-volatile metabolites involved in constitutive defense against herbivores or protection against abiotic stress (Chadwick et al., 2026). For example, high-elevation conditions appear to select willows for higher concentrations and richness of flavonoids, while these compounds remain structurally similar, resulting in low variation among species and individuals (Volf et al., 2023, 2022). Whether the remaining variation in VOC profiles provides ecological benefits to high-elevation willows remains to be tested experimentally. Future studies could examine whether this variation helps maintain interactions with natural enemies and supports VOC-mediated indirect defense under high-elevation conditions.

We observed pronounced differences in VOC profiles between the two willow species pairs. In willows, phylogenetic relatedness often shows little correlation with chemical profiles (Leong et al., 2024; Volf et al., 2015). A similar pattern occurs in VOCs, where unrelated species may share induced blends, whereas closely related species can differ substantially (Mezzomo et al., 2024). The highly variable VOC chemistry of closely related willows may help reduce overlap in their herbivore communities and facilitate the attraction of specific natural enemies (Inui et al., 2003; Mezzomo et al., 2026). Consistent with pattern, *S. mielichhoferi* and *S. myrsinifolia* differed markedly in chemistry despite their close relatedness and morphological similarity, highlighting the potential role of chemical traits in ecological differentiation among sister species (Wang et al., 2022). Additionally, both species are hexaploid and likely of allopolyploid origin (Wagner et al., 2021), which could promote stronger chemical divergence among them compared to the diploid *S. appendiculata-caprea* pair (Edger et al., 2015).

In contrast to the elevational patterns in VOCs, we found no significant elevational trend in predation rates. This contrasts with global patterns along elevational gradients (Zvereva and Kozlov, 2022), especially in tropical systems where predation typically declines with elevation or peaks at mid elevations (Barlow et al., 2024; Camacho and Avilés, 2019; Mrazova and Sam, 2019). In the tropics, these patterns are largely driven by shifts in predator composition, particularly ants dominating at low elevations and birds at mid and high elevations (Camacho and Avilés, 2019; Sam et al., 2015; Mrazova and Sam, 2019). In our study, we recorded no predation by ants, which may explain the absence of a clear elevational trend in invertebrate predation. Insectivorous birds in the Alps occur across broad elevational ranges, although species feeding on vegetation decline above the tree line (Chamberlain et al., 2016; Klosius, 2008). At local scales, the effects of elevation on predation along temperate elevational gradients are also often weaker than the effects of habitat structure, climatic conditions unrelated to elevation, or composition of local predator assemblages (Dean et al., 2024; Cordeiro Pereira et al., 2025). This could potentially explain our results, in which plant species and prey color exerted stronger effects than elevation.

We expected weaker differences in predation among differently colored caterpillar at higher elevations, assuming reduced predator specialization and more generalist foraging (Sam et al., 2024). Contrary to this prediction, color specific predation did not significantly change with elevation in either species pair. Still, yellow caterpillars experienced lower predation at higher elevations in the *S. mielichhoferi–myrsinifolia* pair. These patterns may reflect turnover in predator communities and associated shifts in prey preferences. Although insectivorous birds occur from lowlands to high elevations in the Alps, their community composition changes markedly with elevation (Chamberlain et al., 2016; Klosius, 2008). Birds also respond strongly to caterpillar color and often avoid yellow hues (Hernández-Agüero et al., 2020). Reduced predation on yellow caterpillars at higher elevations may reflect the greater visibility of bright colors in open habitats above the tree line. Alternatively, predators in colder and energetically constrained environments may avoid potentially aposematically colored prey (Piersma, 2002; Sentis et al., 2012) although many yellowish caterpillars without known toxicity occur on willows (Volf et al., 2015).

Overall, between-plant variability in bird predation did not change significantly with elevation, and the two species pairs showed contrasting trends at the highest elevations. In both pairs, however, variability in bird predation tended to increase from low to mid elevations. This pattern may reflect greater heterogeneity in habitat structure near the tree line (Cordeiro Pereira et al., 2025), as well as higher richness or greater variability in insectivorous bird communities at mid elevations (Klosius, 2008). In contrast, between plant variability in invertebrate predation increased till 1250m elevation in *S. appendiculata–caprea* pair and till mid elevation in *S. mielichhoferi–myrsinifolia* pair which declined at higher elevations in both species pairs. This pattern is consistent with tighter thermal constraints on ectotherm activity. As temperatures decrease, windows for invertebrate foraging narrows, making attacks more predictable and synchronized among nearby plants (Kirchner et al., 2025; Sunday et al., 2019). This effect is likely strongest above the tree line, where plant level microclimates track ambient conditions more closely than in forests at lower regions along the gradient (Kirchner et al., 2025).

We detected significant correlations between predation rates and both VOC richness and total VOC emissions. These relationships differed between the two species pairs, suggesting that although birds and invertebrate predators respond to VOC cues (e.g., Amo et al., 2013; Turlings and Erb, 2018), the strength and direction of these responses may depend on plant species, VOC composition, and other associated traits (Mezzomo et al., 2026). This pattern highlights the importance of specificity in induced VOC responses for predator attraction. Importantly, these correlations remained significant after accounting for elevation, indicating that VOCs were associated with predation patterns independently of their elevational trends. Because these relationships are correlative, however, we cannot exclude additional mechanisms acting alongside VOCs. These may include differences in visual cues, such as leaf reflectance, microhabitat conditions, such as exposure and wind, or variation in local predator community composition (Mäntylä et al., 2008; 2020; Klosius, 2008; Barlow et al., 2024).

Predation rates also correlated with emissions of several individual VOCs, including (*E*)-3-hexen-1-ol acetate, β-bourbonene, and α-muurolene, which are commonly induced by herbivory (Du et al., 1998; Klimm et al., 2020; Mezzomo et al., 2023). (*E*)-3-hexen-1-ol acetate occurs in VOC blends that attract insect parasitoids, and isomers of this compound show strong attractant effects on parasitoids and predators (Faal et al., 2021; Shiojiri et al., 2006). β-bourbonene, and α-muurolene are also emitted in response to herbivory (Mezzomo et al., 2023). Both compounds are sesquiterpenes widely known for their roles in defense against insect herbivores and the attraction of predators. However, both β-bourbonene and α-muurolene need further individual testing for their ecological functions (Turlings and Erb, 2018; Klimm et al., 2020). Interestingly, although total VOC emissions decreased with elevation, the emissions of these specific compounds showed no elevational trend. This may indicate that VOCs involved in multitrophic interactions are less affected by environmental conditions than other inducible volatiles. The absence of elevational changes in these compounds could explain why VOCs correlated with predator attraction across elevations. However, experimental verification would be required to confirm whether these metabolites directly attract predators in this system.

Overall, our results show that predation rates were not strongly affected by elevation but correlated with VOC richness, total emissions, and key individual VOCs known to attract predators. Although total VOC emissions significantly or marginally decreased with elevation, emission levels of individual VOCs most strongly associated with predation remained unchanged throughout the elevation. This pattern is consistent with the idea that high elevation environments favor production of metabolites providing the greatest ecological benefits (Defossez et al., 2021; Volf et al., 2023). If similar prioritization applies to VOCs functioning as indirect defenses, it may represent an additional mechanism by which willows, and potentially other alpine plants, optimize defensive strategies across heterogeneous environments. In this system, the ecological role of VOCs may also be reinforced by the relatively weak elevational decline in predation compared with many other gradients. Future studies should test whether plant investment in VOC production varies among environments in relation to predator abundance and predator specialization.

## Data Availability

The datasets presented in this study can be found in online repositories. The names of the repository/repositories and accession number(s) can be found below: https://figshare.com/, https://doi.org/10.6084/m9.figshare.32151942.
