# Supplementary material for: Scents on the slope: declining volatile emissions but stable predator-associated cues across elevation in alpine willows
Source: Front Plant Sci. 2026 Jul 29;17:1873253. doi: 10.3389/fpls.2026.1873253 (PMC13462214; doi:10.3389/fpls.2026.1873253)
Supplement: Supplementary file 1 [file Table1.docx]

Supplementary Material

Scents on the slope: Declining volatile emissions but stable predator-associated cues across elevation in alpine willows

# 1 Molecular identification

We extracted total genomic DNA from dried leaves of each studied individual using the MagAttract HMW DNA kit (Qiagen, USA) following the manufacturer's instructions. The quantity and quality of the DNA were verified using Qubit Fluorometer 3.0 (High Sensitivity Kit; Life Technologies) and on a 2% agarose gel. The extracted genomic DNA was used for library preparation, and sequencing was performed by Novogene (Cambridge, United Kingdom). A total amount of 1.0μg DNA per sample was used as input material for preparation of the Illumina libraries. The genomic DNA was sheared to a size of 350bp, and then the fragments were end-repaired, A-tailed, and ligated with the NEBNext adapter for Illumina sequencing. P5 and indexed P7 oligos were added to the resulting DNA fragments. The resulting libraries were purified using the AMPure XP system (Beckman Coulter, USA) and their size distribution was checked using the Agilent 2100 Bioanalyzer (Agilent Technologies, USA) and quantified by real-time PCR (to meet the minimum input criteria of 3 nM). A whole genome shotgun sequencing was performed on a Novaseq 6000 platform, producing 2x150bp paired end reads.

Ten individuals of each *Salix* spp. – reliably identified based on their morphology – were used in comparative repeat analysis using the RepeatExplorer2 pipeline following Novák *et al.* (2020). Raw Illumina reads were filtered and trimmed to 100 bp using cutadapt 1.15 (Martin, 2011) with options ‘--nextseq-trim=10 --length=100 --minimum-length=100’ supplied with a custom list of the Illumina TruSeq Universal Adapter and Indexed Adapters 1–27. The resulting filtered reads in fastq format were then pseudo-randomly sampled, interleaved and converted to fasta format using seqtk (ver. 1.2; https://github.com/lh3/seqtk). We sampled and compared ca 0.55x equivalent of the genome size of each *S. appendiculata* and *S. caprea,* and a 0.3x equivalent of the genome size of each *S. myrsinifolia* and *S. mielichhoferi*. We analysed 2–4 individuals per species, one analysis per individual. From each individual, about 0.25 cm^2^ of a leaf was chopped with a similar amount of the leaf of the internal standard (*Solanum pseudocapsicum*, 1C = 1.295; Temsch *et al.*, 2010) in 400 mL of the Otto I buffer. The suspension was filtered through a 42-μm nylon mesh, incubated for ca 2 min and stained with Otto II buffer containing 2-mercaptoethanol (2 μL / mL) and propidium iodide (50 μg / mL) for ca 5 min. The samples were analysed using a Sysmex CyFlow Space flow cytometer equipped with a solid-state green (532 nm) laser. Fluorescence of at least 5,000 events was recorded, and the mean fluorescence of the sample and the standard G1 peak were calculated. The estimated genome sizes were 1C = 419 Mbp for *S. appendiculata*, 1C = 451 Mbp for *S. caprea*, 1C = 1,480 Mbp for *S. mielichhoferi* and 1C = 1,091 Mbp for *S. myrsinifolia*. This confirms diploid levels for *S. appendiculata* and *S. caprea*, and hexaploid levels for *S. mielichhoferi* and *S. myrsinifolia* (Wagner et al. 2021). The read IDs of the resulting samples were tagged, concatenated, and analysed together using RepeatExplorer2 with TAREAN (Singularity container tarean:0.3.12-7a7dc9e) with options ‘--automatic_filtering --mincl 0.001 --assembly_min 3 --taxon VIRIDIPLANTAE4.0’. We then used linear models with empirical Bayes moderation and Benjamini-Hochberg correction fitted in the ‛limma՚ R package (Ritchie *et al.*, 2015) to identify the read clusters best separating the reliably identified *S. appendiculata* from *S. caprea* individuals and *S. myrsinifolia* individuals from *S. mielichhoferi* individuals. We used 50 best clusters for *S. appendiculata* and *S. caprea* and 100 best clusters for S *mielichhoferi* and *S. myrsinifolia,* which have a larger genome size than the first species pair.

Consensus sequences of these identified diagnostic repeat clusters were merged into a single FASTA file; TAREAN consensus dimer sequences were used where available for identified tandem repeats. The resulting reference was indexed and used for mapping of filtered (see above) Illumina sequencing data of all sampled individuals using bowtie2 (Langmead and Salzberg, 2012) with options '-N 1 -X 101'. We used samtools view 1.9. (Danecek *et al.*, 2021) with flag ‘-F 256’ to filter only primary alignments. The resulting bam files were sorted using samtools sort and used to calculate per base read depth using bedtools genomecov with options '-d -ibam' (Quinlan and Hall, 2010). The read depth was summed across the repeat clusters using bedtools group by with option ‘-o sum’. Finally, we normalised the number of reads per diagnostic repeat clusters of each individual based on their respective total amount of filtered Illumina sequencing data used for mapping. We performed Principal Component Analysis (PCA) in Canoco 5 (ter Braak and Smilauer, 2012) to visualise how the individual samples clustered. We performed separate analyses for *S. appendiculata* and *S. caprea* and for *S. mielichhoferi* and *S. myrsinifolia*.

Figure S1. PCA plot showing the clustering of studied willow individuals based on the number of diagnostic repetitive repeat clusters for *S. appendiculata* and *S. caprea*. Individuals are represented as circles or squares. Those reliably identified based on morphology and used to define diagnostic repeat clusters are shown in dark blue (*S. appendiculata*) and dark red (*S. caprea*). Other individuals with clear morphological traits of one of the two species are shown in light blue (*S. appendiculata*) and light red (*S. caprea*). Four individuals exhibiting intermediate morphology are shown in orange. Two of these clustered within the *S. appendiculata* group, and we treated them as such in our subsequent analyses. Ovals delineate individuals we considered to be *S. appendiculata* and *S. caprea*. The remaining two individuals with intermediate morphology, located outside these ovals, were tentatively identified as potential hybrids and excluded from further analyses.

In the case of *S. appendiculata* and *S. caprea*, the first two PCA axes explained 50.97% of the variation in read number for the diagnostic repeat clusters (Figure S1). Individuals reliably identified based on morphology (that we used to select the diagnostic repeat clusters) clustered with others exhibiting the typical morphology of the respective species. The only exception was individual ALP047, which appeared as an outlier within the *S. appendiculata* group. However, ALP047 displayed clear *S. appendiculata* morphology with no characteristic traits of *S. caprea* or related species, and it was distant from the *S. caprea* cluster. Therefore, we retained it as *S. appendiculata*. Four individuals exhibited intermediate morphology between *S. appendiculata* and *S. caprea*. Two of them were grouped within the *S. appendiculata* cluster and were considered *S. appendiculata* for subsequent analyses. The remaining two individuals (ALP018 and ALP031), positioned between the *S. appendiculata* and *S. caprea* clusters, were tentatively identified as potential hybrids. However, given their small number, it was not meaningful to treat potential hybrids as a separate category. Consequently, we excluded these two individuals from further analyses.

Figure S2. PCA plot showing the clustering of studied willow individuals based on the number of diagnostic repeat clusters for *S. mielichhoferi* and *S. myrsinifolia*. Individuals are represented as circles. Those reliably identified based on morphology and used to define diagnostic repeat clusters are shown in dark purple (*S. mielichhoferi*) and dark green (*S. myrsinifolia*). Other individuals with clear morphological traits of one of the two species are shown in light purple (*S. mielichhoferi*) and light green (*S. myrsinifolia*). Individuals with a clearly intermediate morphology are in orange and individuals that could not be reliably assigned to any category based on their morphology are in grey. Ovals delineate individuals we considered to be *S. mielichhoferi* and *S. myrsinifolia* in our following analyses. The remaining three individuals located outside these ovals were tentatively identified as potential hybrids with other willow species and excluded from further analyses.

In the case of *S. mielichhoferi* and *S. myrsinifolia*, the first two PCA axes explained 77.36% of the variation in read counts for the diagnostic repeat clusters (Figure S2). Individuals reliably identified based on morphology (that we used to define the diagnostic repeat clusters) and others exhibiting typical morphology of the respective species formed two distinct, though less clearly separated, clusters compared to the previous system, reflecting the close relatedness of these species. Individuals with intermediate morphology, as well as some that could not be reliably assigned to a species based on morphology, were distributed across both clusters. These individuals were classified as either *S. mielichhoferi* or *S. myrsinifolia* in subsequent analyses according to their PCA placement. Three individuals, ALP049 with intermediate morphology and ALP098 plus ALP138 with unclear morphological identification, were positioned outside the main clusters. These were considered potential hybrids with other willow species and were excluded from further analyses.

**References**

Danecek, P., Bonfield, J. K., Liddle, J., Marshall, J., Ohan, V., Pollard, M.O., Whitwham, A., Keane, T., McCarthy, S. A., & Davies, R.M. (2021). Twelve years of SAMtools and BCFtools. *Gigascience*, *10*(2), 1-4. <https://doi.org/10.1093/gigascience/giab008>

Langmead, B., & Salzberg, S. L. (2012). Fast gapped-read alignment with Bowtie 2. *Nat. Methods*, 9, 357-359. doi:10.1038/nmeth.192

Martin, M. (2011). Cutadapt removes adapter sequences from high-throughput sequencing reads. *EMBnet. J.*, 17, 10-12. <https://doi.org/10.14806/ej.17.1.200>

Novák, P., Neumann, P., & Macas, J. (2020). Global analysis of repetitive DNA from unassembled sequence reads using RepeatExplorer2. *Nat. Protoco.* 15, 3745-3776. https://doi.org/10.1038/s41596-020-0400-y

Quinlan, A. R., & Hall, I. M. (2010). BEDTools: A flexible suite of utilities for comparing genomic features. *Bioinformatics*, 26, 841-842. https://doi.org/10.1093/bioinformatics/btq033

Ritchie, M. E, Phipson, B., Wu, D., Hu, Y., Law, C. W., Shi, W., & Smyth, G. K. (2015). limma powers differential expression analyses for RNA-sequencing and microarray studies. *Nucleic Acids Res*, 43, e47-e47. https://doi.org/10.1093/nar/gkv007

Temsch, E. M., Greilhuber, J., & Krisai, R. (2010). Genome size in liverworts. *Preslia,* *82*(1), 63–80.

ter Braak, C. J., & Smilauer, P. (2012). Canoco reference manual and user’s guide: Software for ordination (version 5.0). Ithaca: Microcomputer power.
