# Supplementary material for: Scents on the slope: declining volatile emissions but stable predator-associated cues across elevation in alpine willows
Source: Front Plant Sci. 2026 Jul 29;17:1873253. doi: 10.3389/fpls.2026.1873253 (PMC13462214; doi:10.3389/fpls.2026.1873253)
Supplement: Supplementary file 2 [file Table2.docx]

Supplementary Figures

**Scents on the slope: Declining volatile emissions but stable predator-associated cues across elevation in alpine willows**

**Figure S3.** Field sampling and experimental setup. Left: Procedure for collecting volatile organic compounds (VOCs), in which willow branches were enclosed in polyamide bags. The enlarged view shows the VOC-adsorbing material (two polydimethylsiloxane (PDMS) tubes) positioned on branches under field conditions. Right: Predation experiment using artificial caterpillars in three colours (green, brown, and yellow). For each willow individual, three branches were selected, with each branch receiving two caterpillars of a single colour. The allocation of colours to branches was randomised among plants. A 5 mM methyl jasmonate (MeJA) aqueous solution was sprayed before VOC sampling and after caterpillar placement to induce VOC emission. Figure created in Biorender.com and modified.

**Figure S4.** A Venn diagram showing the number of unique and shared VOCs between the four willow species. A total of 31 VOCs were common in all four species. Among the four species, *Salix caprea* showed the highest VOC richness (40 compounds), followed by *S. mielichhoferi* (39), *S. myrsinifolia* (34) and *Salix appendiculata* (35). The *S.* *appendiculata*-*caprea* pair emitted 40 VOCs, whereas the *S*. *mielichhoferi*-*S*. *myrsinifolia* pair emitted 39 VOCs in total.

**Figure S5**. Elevational trends in variability of VOC emissions and richness. (A) In the *S. appendiculata–caprea* pair, VOC variability differed among elevations (PERMDISP; F(7,44) = 4.132, p = 0.003) showing non-linear decreasing trend (GAM: χ²(1.99) = 0.046, p = 0.001). (B) In the *S. mielichhoferi–myrsinifolia* pair, no elevational change in VOC variability was found (PERMDISP; F(11,38) = 1.678, p = 0.128). Significant results are marked with asterisks (*) and non-significant results are with “n.s.” (= non-significant) on the top right corner and dashed trend lines.

**Figure S6**. Elevational trends in predation on green, brown and yellow caterpillars as analysed with a Generalised Linear Model (GLM). (A) In the *S. appendiculata–caprea* species pair, no significant elevational differences were found. (B) In the *S. mielichhoferi–myrsinifolia* species pair, elevation affected the predation of yellow caterpillars, which experienced significantly lower predation at higher elevations (Nagelkerke R² = 0.82, LR χ²(5) = 451.0, p < 0.001). Significant results are marked with asterisks (*) and non-significant results are with “n.s.” (= non-significant) on the top right corner and dashed trend lines.
