# Supplementary material for: Scents on the slope: declining volatile emissions but stable predator-associated cues across elevation in alpine willows
Source: Front Plant Sci. 2026 Jul 29;17:1873253. doi: 10.3389/fpls.2026.1873253 (PMC13462214; doi:10.3389/fpls.2026.1873253)
Supplement: Supplementary file 3 [file Table3.docx]

Supplementary Tables

**Scents on the slope: Declining volatile emissions but stable predator-associated cues across elevation in alpine willows**

# Table S1. An overview of the study sites, including elevation (m asl), GPS coordinates, number of plants from our focal species (based on molecular identifications), and total number of plants sampled per site.

| Sites  Name | Elevation  (m) | GPS Coordinates | *Salix appendiculata* | *Salix caprea* | *Salix mielichhoferi* | *Salix myrsinifolia* | Total  Trees |
| --- | --- | --- | --- | --- | --- | --- | --- |
| S5 | 1250 | 47.0389328N, 12.8357181E | 5 | 4 | 1 | 3 | 13 |
| S10 | 1850 | 47.0607244N, 12.8328175E | 4 | 0 | 0 | 0 | 4 |
| S21 | 1650 | 47.0338717N, 12.7932111E | 4 | 0 | 8 | 0 | 12 |
| S23 | 1850-1860 | 47.0632417N, 12.8194747E | 5 | 2 | 3 | 0 | 10 |
| S24 | 1100 | 47.0274942N, 12.8566869E | 8 | 0 | 0 | 4 | 12 |
| S26 | 1050 | 46.9922925N, 12.8753158E | 3 | 5 | 0 | 5 | 13 |
| S27 | 1000 | 46.9733794N, 12.8892111E | 3 | 5 | 0 | 4 | 12 |
| S29 | 1300 | 47.0473886N, 12.8092917E | 0 | 0 | 5 | 0 | 5 |
| S35 | 2230 | 47.0812233N, 12.7652661E | 0 | 0 | 5 | 0 | 5 |
| S36 | 2050 | 47.0707594N, 12.7651033E | 0 | 0 | 5 | 0 | 5 |
| S37 | 1910 | 47.0542500N, 12.8334936E | 0 | 0 | 1 | 0 | 1 |
| S38 | 2050-2150 | 47.04288N,  12.46092E | 4 | 0 | 6 | 0 | 10 |

# Table S2. Details on the studied willow individuals. The table shows their species identity, elevation, site, total no. of caterpillars checked, number of predation events, potential predation per sample (i.e. total checked caterpillars multiplied by number of observation) VOC richness, and total VOC emissions. Total VOC emissions is measured in total ion count (TIC).

| Willow individual | | Species | | Elevation (m) | | Site | | Total Caterpillars  Checked | | Predation Events | | Potential  Predation | | VOC Richness | | Total VOC Emissions (TIC) | |  |
| --- | --- | --- | --- | --- | --- | --- | --- | --- | --- | --- | --- | --- | --- | --- | --- | --- | --- | --- |
|  | ALP_001 | | *Salix caprea* | | 1000 | | S27 | | 11 | | 11 | | 22 | | 14 | | 8752449.772 | |
|  | ALP_002 | | *Salix caprea* | | 1000 | | S27 | | 12 | | 11 | | 24 | | 11 | | 14796885.82 | |
|  | ALP_003 | | *Salix caprea* | | 1000 | | S27 | | 12 | | 16 | | 24 | | 12 | | 8846729.587 | |
|  | ALP_004 | | *Salix myrsinifolia* | | 1000 | | S27 | | 12 | | 13 | | 24 | | 13 | | 3229320.502 | |
|  | ALP_005 | | *Salix caprea* | | 1000 | | S27 | | 12 | | 12 | | 24 | | 16 | | 10078128.1 | |
|  | ALP_006 | | *Salix caprea* | | 1000 | | S27 | | 11 | | 8 | | 22 | | 12 | | 7281726.253 | |
|  | ALP_008 | | *Salix myrsinifolia* | | 1000 | | S27 | | 12 | | 8 | | 24 | | 13 | | 9336185.033 | |
|  | ALP_011 | | *Salix myrsinifolia* | | 1000 | | S27 | | 12 | | 9 | | 24 | | 12 | | 5971073.183 | |
|  | ALP_012 | | *Salix myrsinifolia* | | 1000 | | S27 | | 12 | | 9 | | 24 | | 9 | | 3031279.118 | |
|  | ALP_014 | | *Salix appendiculata* | | 1000 | | S27 | | 12 | | 3 | | 24 | | 11 | | 3698036.632 | |
|  | ALP_015 | | *Salix appendiculata* | | 1000 | | S27 | | 12 | | 4 | | 24 | | 13 | | 7862255.382 | |
|  | ALP_016 | | *Salix appendiculata* | | 1000 | | S27 | | 12 | | 6 | | 24 | | 13 | | 13193596.56 | |
|  | ALP_017 | | *Salix caprea* | | 1050 | | S26 | | 12 | | 9 | | 24 | | 15 | | 10103766.55 | |
|  | ALP_019 | | *Salix caprea* | | 1050 | | S26 | | 12 | | 12 | | 24 | | 12 | | 3128245.764 | |
|  | ALP_020 | | *Salix caprea* | | 1050 | | S26 | | 12 | | 5 | | 24 | | 14 | | 5071742.501 | |
|  | ALP_021 | | *Salix myrsinifolia* | | 1050 | | S26 | | 10 | | 12 | | 20 | | 9 | | 3523898.538 | |
|  | ALP_022 | | *Salix myrsinifolia* | | 1050 | | S26 | | 12 | | 5 | | 24 | | 14 | | 12122699.45 | |
|  | ALP_023 | | *Salix appendiculata* | | 1050 | | S26 | | 12 | | 2 | | 24 | | 12 | | 19975459.6 | |
|  | ALP_024 | | *Salix myrsinifolia* | | 1050 | | S26 | | 12 | | 7 | | 24 | | 4 | | 2371676.687 | |
|  | ALP_025 | | *Salix myrsinifolia* | | 1050 | | S26 | | 11 | | 6 | | 22 | | 12 | | 4717223.861 | |
|  | ALP_026 | | *Salix appendiculata* | | 1050 | | S26 | | 12 | | 9 | | 24 | | 17 | | 16213926.2 | |
|  | ALP_027 | | *Salix appendiculata* | | 1050 | | S26 | | 12 | | 8 | | 24 | | 20 | | 20246127.16 | |
|  | ALP_028 | | *Salix myrsinifolia* | | 1050 | | S26 | | 12 | | 9 | | 24 | | 19 | | 11521335.93 | |
|  | ALP_029 | | *Salix caprea* | | 1050 | | S26 | | 12 | | 7 | | 24 | | 13 | | 10763797.98 | |
|  | ALP_030 | | *Salix caprea* | | 1050 | | S26 | | 12 | | 6 | | 24 | | 26 | | 21894168.11 | |
|  | ALP_035 | | *Salix appendiculata* | | 1100 | | S24 | | 12 | | 2 | | 24 | | 17 | | 22168326.53 | |
|  | ALP_036 | | *Salix appendiculata* | | 1100 | | S24 | | 12 | | 1 | | 24 | | 18 | | 14361304.45 | |
|  | ALP_037 | | *Salix myrsinifolia* | | 1100 | | S24 | | 12 | | 4 | | 24 | | 20 | | 8771725.461 | |
|  | ALP_038 | | *Salix appendiculata* | | 1100 | | S24 | | 12 | | 2 | | 24 | | 20 | | 14957010.02 | |
|  | ALP_039 | | *Salix myrsinifolia* | | 1100 | | S24 | | 12 | | 2 | | 24 | | 17 | | 7229748.652 | |
|  | ALP_040 | | *Salix appendiculata* | | 1100 | | S24 | | 12 | | 7 | | 24 | | 18 | | 22885176.03 | |
|  | ALP_041 | | *Salix appendiculata* | | 1100 | | S24 | | 12 | | 8 | | 24 | | 19 | | 22794215.73 | |
|  | ALP_042 | | *Salix appendiculata* | | 1100 | | S24 | | 12 | | 2 | | 24 | | 9 | | 9495444.858 | |
|  | ALP_043 | | *Salix appendiculata* | | 1100 | | S24 | | 12 | | 6 | | 24 | | 14 | | 17968173.73 | |
|  | ALP_044 | | *Salix appendiculata* | | 1100 | | S24 | | 12 | | 2 | | 24 | | 14 | | 17408022.52 | |
|  | ALP_045 | | *Salix myrsinifolia* | | 1100 | | S24 | | 12 | | 1 | | 24 | | 22 | | 5536458.887 | |
|  | ALP_046 | | *Salix myrsinifolia* | | 1100 | | S24 | | 12 | | 6 | | 24 | | 20 | | 2391320.759 | |
|  | ALP_047 | | *Salix appendiculata* | | 1250 | | S5 | | 12 | | 4 | | 24 | | 14 | | 10201230.44 | |
|  | ALP_048 | | *Salix myrsinifolia* | | 1250 | | S5 | | 12 | | 9 | | 24 | | 21 | | 4665168.273 | |
|  | ALP_050 | | *Salix appendiculata* | | 1250 | | S5 | | 12 | | 2 | | 24 | | 12 | | 6067024.292 | |
|  | ALP_051 | | *Salix appendiculata* | | 1250 | | S5 | | 12 | | 0 | | 24 | | 15 | | 10102759.25 | |
|  | ALP_052 | | *Salix myrsinifolia* | | 1250 | | S5 | | 12 | | 5 | | 24 | | 23 | | 22335670.8 | |
|  | ALP_053 | | *Salix mielichhoferi* | | 1250 | | S5 | | 12 | | 4 | | 24 | | 25 | | 26015110.28 | |
|  | ALP_054 | | *Salix appendiculata* | | 1250 | | S5 | | 12 | | 6 | | 24 | | 12 | | 11498425.3 | |
|  | ALP_055 | | *Salix appendiculata* | | 1250 | | S5 | | 12 | | 1 | | 24 | | 8 | | 4414639.252 | |
|  | ALP_056 | | *Salix myrsinifolia* | | 1250 | | S5 | | 12 | | 4 | | 24 | | 19 | | 11540659.88 | |
|  | ALP_057 | | *Salix caprea* | | 1250 | | S5 | | 12 | | 2 | | 24 | | 21 | | 27486810.76 | |
|  | ALP_058 | | *Salix caprea* | | 1250 | | S5 | | 12 | | 4 | | 24 | | 15 | | 4244020.148 | |
|  | ALP_059 | | *Salix caprea* | | 1250 | | S5 | | 12 | | 12 | | 24 | | 18 | | 12382973.62 | |
|  | ALP_060 | | *Salix caprea* | | 1250 | | S5 | | 12 | | 8 | | 24 | | 6 | | 2421835.438 | |
|  | ALP_067 | | *Salix appendiculata* | | 1850 | | S10 | | 12 | | 8 | | 24 | | 17 | | 5893683.143 | |
|  | ALP_068 | | *Salix appendiculata* | | 1850 | | S10 | | 12 | | 9 | | 24 | | 14 | | 10860441.63 | |
|  | ALP_070 | | *Salix appendiculata* | | 1850 | | S10 | | 12 | | 7 | | 24 | | 15 | | 3573104.876 | |
|  | ALP_071 | | *Salix appendiculata* | | 1850 | | S10 | | 12 | | 7 | | 24 | | 19 | | 9862960.668 | |
|  | ALP_078 | | *Salix appendiculata* | | 1850 | | S23 | | 12 | | 7 | | 24 | | 12 | | 10937204.34 | |
|  | ALP_079 | | *Salix appendiculata* | | 1850 | | S23 | | 12 | | 6 | | 24 | | 18 | | 12766815.37 | |
|  | ALP_080 | | *Salix appendiculata* | | 1850 | | S23 | | 12 | | 11 | | 24 | | 10 | | 9881040.741 | |
|  | ALP_081 | | *Salix mielichhoferi* | | 1850 | | S23 | | 11 | | 3 | | 22 | | 25 | | 21997256.88 | |
|  | ALP_082 | | *Salix appendiculata* | | 1850 | | S23 | | 12 | | 11 | | 24 | | 10 | | 9838102.789 | |
|  | ALP_083 | | *Salix appendiculata* | | 1850 | | S23 | | 12 | | 6 | | 24 | | 14 | | 12363929.22 | |
|  | ALP_084 | | *Salix mielichhoferi* | | 1850 | | S23 | | 12 | | 8 | | 24 | | 21 | | 21020684.2 | |
|  | ALP_086 | | *Salix mielichhoferi* | | 1300 | | S29 | | 11 | | 2 | | 22 | | 22 | | 23858431.22 | |
|  | ALP_087 | | *Salix mielichhoferi* | | 1300 | | S29 | | 12 | | 1 | | 24 | | 18 | | 17901303.23 | |
|  | ALP_088 | | *Salix mielichhoferi* | | 1300 | | S29 | | 12 | | 5 | | 24 | | 32 | | 30098591.55 | |
|  | ALP_089 | | *Salix mielichhoferi* | | 1300 | | S29 | | 12 | | 4 | | 24 | | 29 | | 20939524.48 | |
|  | ALP_090 | | *Salix mielichhoferi* | | 1300 | | S29 | | 12 | | 5 | | 24 | | 22 | | 8669118.308 | |
|  | ALP_091 | | *Salix mielichhoferi* | | 1650 | | S21 | | 12 | | 1 | | 24 | | 28 | | 19979209.64 | |
|  | ALP_092 | | *Salix mielichhoferi* | | 1650 | | S21 | | 12 | | 6 | | 24 | | 26 | | 16992863.19 | |
|  | ALP_093 | | *Salix appendiculata* | | 1650 | | S21 | | 12 | | 8 | | 24 | | 14 | | 6578099.192 | |
|  | ALP_094 | | *Salix mielichhoferi* | | 1650 | | S21 | | 12 | | 2 | | 24 | | 20 | | 13441213.35 | |
|  | ALP_095 | | *Salix appendiculata* | | 1650 | | S21 | | 12 | | 5 | | 24 | | 11 | | 4822425.676 | |
|  | ALP_096 | | *Salix mielichhoferi* | | 1650 | | S21 | | 12 | | 4 | | 24 | | 24 | | 20466599.16 | |
|  | ALP_097 | | *Salix appendiculata* | | 1650 | | S21 | | 12 | | 7 | | 24 | | 11 | | 5622373.388 | |
|  | ALP_099 | | *Salix mielichhoferi* | | 1650 | | S21 | | 12 | | 8 | | 24 | | 24 | | 22684869.98 | |
|  | ALP_100 | | *Salix mielichhoferi* | | 1650 | | S21 | | 12 | | 8 | | 24 | | 24 | | 5867374.755 | |
|  | ALP_101 | | *Salix appendiculata* | | 1650 | | S21 | | 12 | | 5 | | 24 | | 9 | | 8860909.477 | |
|  | ALP_102 | | *Salix mielichhoferi* | | 1650 | | S21 | | 12 | | 7 | | 24 | | 20 | | 11596705.06 | |
|  | ALP_103 | | *Salix mielichhoferi* | | 1650 | | S21 | | 11 | | 5 | | 22 | | 24 | | 14001051.12 | |
|  | ALP_110 | | *Salix mielichhoferi* | | 2050 | | S36 | | 12 | | 2 | | 24 | | 27 | | 12039516.55 | |
|  | ALP_111 | | *Salix mielichhoferi* | | 2050 | | S36 | | 12 | | 3 | | 24 | | 19 | | 10285680.65 | |
|  | ALP_112 | | *Salix mielichhoferi* | | 2050 | | S36 | | 12 | | 3 | | 24 | | 21 | | 16773031.26 | |
|  | ALP_113 | | *Salix mielichhoferi* | | 2050 | | S36 | | 12 | | 4 | | 24 | | 20 | | 30767259.19 | |
|  | ALP_114 | | *Salix mielichhoferi* | | 2050 | | S36 | | 12 | | 3 | | 24 | | 20 | | 17260558.51 | |
|  | ALP_123 | | *Salix mielichhoferi* | | 2050 | | S38 | | 12 | | 3 | | 24 | | 18 | | 17245684.82 | |
|  | ALP_124 | | *Salix mielichhoferi* | | 2230 | | S35 | | 12 | | 4 | | 24 | | 18 | | 7798061.649 | |
|  | ALP_125 | | *Salix mielichhoferi* | | 2230 | | S35 | | 12 | | 5 | | 24 | | 18 | | 4609738.957 | |
|  | ALP_126 | | *Salix mielichhoferi* | | 2230 | | S35 | | 12 | | 5 | | 24 | | 28 | | 12448978.8 | |
|  | ALP_127 | | *Salix mielichhoferi* | | 2230 | | S35 | | 12 | | 7 | | 24 | | 24 | | 8668683.99 | |
|  | ALP_128 | | *Salix mielichhoferi* | | 2230 | | S35 | | 12 | | 5 | | 24 | | 20 | | 11883397.96 | |
|  | ALP_129 | | *Salix mielichhoferi* | | 2150 | | S38 | | 12 | | 6 | | 24 | | 16 | | 5780044.329 | |
|  | ALP_130 | | *Salix appendiculata* | | 2150 | | S38 | | 12 | | 2 | | 24 | | 6 | | 5486725.896 | |
|  | ALP_131 | | *Salix appendiculata* | | 2150 | | S38 | | 12 | | 5 | | 24 | | 6 | | 4271567.243 | |
|  | ALP_132 | | *Salix mielichhoferi* | | 2150 | | S38 | | 12 | | 4 | | 24 | | 15 | | 8299127.724 | |
|  | ALP_133 | | *Salix mielichhoferi* | | 2150 | | S38 | | 12 | | 4 | | 24 | | 19 | | 8229439.268 | |
|  | ALP_134 | | *Salix mielichhoferi* | | 2150 | | S38 | | 12 | | 6 | | 24 | | 19 | | 13553213.46 | |
|  | ALP_135 | | *Salix appendiculata* | | 2150 | | S38 | | 12 | | 8 | | 24 | | 5 | | 1957626.356 | |
|  | ALP_136 | | *Salix mielichhoferi* | | 2150 | | S38 | | 12 | | 9 | | 24 | | 15 | | 6629413.769 | |
|  | ALP_139 | | *Salix appendiculata* | | 2150 | | S38 | | 12 | | 11 | | 24 | | 8 | | 2769888.768 | |
|  | ALP_140 | | *Salix mielichhoferi* | | 1860 | | S23 | | 12 | | 5 | | 24 | | 18 | | 9596385.522 | |
|  | ALP_141 | | *Salix caprea* | | 1860 | | S23 | | 12 | | 7 | | 24 | | 19 | | 8859565.63 | |
|  | ALP_142 | | *Salix caprea* | | 1860 | | S23 | | 12 | | 1 | | 24 | | 15 | | 5166071.958 | |
|  | ALP_143 | | *Salix mielichhoferi* | | 1910 | | S37 | | 12 | | 15 | | 24 | | 22 | | 7174938.72 | |

Table S3. Volatile organic compounds (VOCs) emitted by *Salix* *appendiculata*, *S.* *caprea*, *S.* *mielichhoferi*, and *S.* *myrsinifolia* and identified using standards and retention index. Metabolites are listed according to their class. Detected retention time (in minutes), measured retention index calculated with a series of n-alkanes (RI ^a^), retention index based on previous studies using the RTX-Wax/similar column according to the NIST library (RI ^b^), and retention index based on the standards (RI^s^) are shown in the respective columns.

| Compound class | Compound name | RT [min] | RI ^a^ | RI ^b^ | RI^s^ | Ref |
| --- | --- | --- | --- | --- | --- | --- |
| Aldehyde | Nonanal | 5.242 | 1395 | 1394 | 1395 | 1 |
| Alkane | Tetradecane | 5.251 | 1396 | - | 1400 |  |
| Benzenoid | p-cymene | 4.575 | 1281 | 1271 | - | 2 |
| GLV | (*E*)-2-hexenal | 4.215 | 1225 | 1228 | - | 3 |
|  | (*Z*)-3-hexen-1-ol | 5.128 | 1375 | 1377 | 1375 | 4 |
|  | (3*E*)-3-hexenyl butyrate | 5.201 | 1388 | 1454 | - | 5 |
|  | (*Z*)-3-hexen-1-ol acetate | 4.804 | 1318 | 1321 | 1318 | 6 |
| Monoterpene | α-pinene | 2.914 | 1043 | 1020 | 1043 | 7 |
|  | β-pinene | 3.857 | 1172 | 1093 | 1172 | 8 |
|  | β-myrcene | 3.85 | 1173 | 1160 | 1172 | 7 |
|  | Sabinene | 3.582 | 1135 | 1124 | - | 9 |
|  | Limonene | 4.13 | 1212 | 1206 | 1211 | 7 |
|  | Eucalyptol | 4.194 | 1222 | 1227 | 1222 | 7 |
|  | (*E*)-β-ocimene | 4.321 | 1242 | 1241 | 1242 | 8 |
|  | (*Z*)-β-ocimene | 4.432 | 1259 | 1227 | 1259 | 8 |
|  | γ-terpinene | 4.427 | 1259 | 1234 | 1259 | 10 |
|  | Linalool | 6.004 | 1530 | 1530 | 1530 | 11 |
|  | Cosmene | 5.536 | 1448 | 1460 | - | 12 |
| Homoterpene | (*E*)-4,8-dimethylnona-1,3,7-triene | 4.756 | 1309 | 1309 | - | 6 |
| Sesquiterpene | α-copaene | 4.566 | 1510 | 1496 | - | 4 |
|  | α-cubebene | 5.661 | 1470 | 1460 | - | 4 |
|  | (*E*)-β-ocimene epoxide | 5.741 | 1485 | 1492 | - | 13 |
|  | α-guaiene | 5.784 | 1492 | 1591 | - | 14 |
|  | α-ylangene | 5.828 | 1500 | 1485 | - | 15 |
|  | Cyclosativene | 5.852 | 1504 | 1483 | - | 17 |
|  | β-bourbonene | 6.043 | 1537 | 1520 | - | 18 |
|  | (*Z*)-β-copaene | 6.13 | 1552 | 1565 | - | 19 |
|  | (*E)*-β-copaene | 6.376 | 1594 | 1586 | - | 20 |
|  | (*E*)-β-caryophyllene | 6.524 | 1619 | 1620 | 1619 | 21 |
|  | Isogermacrene D | 6.784 | 1660 | 1665 | - | 4 |
|  | Humulene | 6.928 | 1691 | 1690 | 1691 | 22 |
|  | γ-muurolene | 7.074 | 1705 | 1695 | - | 23 |
|  | (*Z*, *E*)-α-farnesene | 7.183 | 1721 | 1721 | 1721 | 24 |
|  | Germacrene D | 7.231 | 1728 | 1710 | - | 18 |
|  | α-muurolene | 7.292 | 1737 | 1716 | - | 4 |
|  | (*E*, *E*)-α-farnesene | 7.349 | 1745 | 1740 | 1745 | 25 |
|  | δ-cadinene | 7.519 | 1769 | 1758 | - | 14 |
|  | γ-cadinene | 7.567 | 1776 | 1760 | - | 14 |
|  | Sesquiterpene 1 | 8.228 | 1966 | - | - | - |
|  | Sesquiterpene 2 | 8.392 | 1988 | - | - | - |
|  | Sesquiterpene 3 | 9.446 | 2156 | - | - | - |
| Jasmonate | Methyl jasmonate | 11.571 | 2259 | 2332 | - | 25 |

# References – Table S3

# 1. Prososki, R. A., Etzel, M. R., and Rankin, S. A. (2007). Solvent type affects the number, distribution, and relative quantities of volatile compounds found in sweet whey powder. *J. Dairy Sci*. 90, 523–531. 10.3168/jds.S0022-0302(07)71535-7

# 2. Paolini, J., Costa, J., and Bernardini, A. (2005). Analysis of the essential oil from aerial parts of Eupatorium cannabinum subsp. corsicum (L.) by gas chromatography with electron impact and chemical ionization mass spectrometry. *J. Chromatogr. A* 1076, 170–178. 10.1016/j.chroma.2005.03.131

# 3. Hallier, A., Prost, C., and Serot, T. (2005). Influence in rearing conditions on the volatile compounds of cooked fillets of Silurus glanis (European catfish). *J. Agric. Food Chem*. 53, 7204–7211. 10.1021/jf050559o

# 4. Paolini, J., Tomi, P., Bernardini, A. F., Bradesi, P., Casanova, J., and Kaloustian, J. (2008). Detailed analysis of the essential oil from Cistus albidus L. by combination of GC/RI, GC/MS and 13C-NMR spectroscopy. *N. Z. J. Agric. Res*. 22, 1270–1278

# 5. Andriamaharavo, N. R. (2014). Retention data. NIST Mass Spectrometry Data Center.

# 6. Paolini, J., Muselli, A., Bernardini, A. F., Bighelli, A., Casanova, J., and Costa, J. (2007). Thymol derivatives from essential oil of Doronicum corsicum L. *Flavour Fragr. J*. 22, 479–487. 10.1002/ffj.1824

# 7. Shellie, R., Mondello, L., Marriott, P., and Dugo, G. (2002). Characterisation of lavender essential oils by using gas chromatography–mass spectrometry with correlation of linear retention indices and comparison with comprehensive two-dimensional gas chromatography. *J. Chromatogr. A* 970, 225–234. 10.1016/S0021-9673(02)00653-2

# 8. Dugo, P., Mondello, L., Zappia, G., Bonaccorsi, I., Cotroneo, A., and Russo, M. T. (2004). The composition of the volatile fraction and the enantiomeric distribution of five volatile components of faustrime oil (Monocitrus australatica × Fortunella sp. × Citrus aurantifolia). *J. Essent. Oil Res*. 16, 328–333. 10.1080/10412905.2004.9698734

# 9. Cavalli, J. F., Ranarivelo, L., Ratsimbason, M., Bernardini, A. F., and Casanova, J. (2001). Constituents of the essential oil of six Helichrysum species from Madagascar. *Flavour Fragr. J.* 16, 253–256. 10.1002/ffj.994

# 10. Shellie, R., Marriott, P., Zappia, G., Mondello, L., and Dugo, G. (2003). Interactive use of linear retention indices on polar and apolar columns with an MS library for reliable characterization of Australian tea tree and other Melaleuca sp. oils. *J. Essent. Oil Res.* 15, 305–312. 10.1080/10412905.2003.9698597

# 11. Kéita, S. M., Vincent, C., Schmit, J. P., and Bélanger, A. (2000). Essential oil composition of Ocimum basilicum L., O. gratissimum L. and O. suave L. in the Republic of Guinea. *Flavour Fragr. J*. 15, 339–341. 10.1002/ffj.922

# 12. Flamini, G., Cioni, P. L., and Morelli, I. (2002). Differences in the fragrances of pollen and different floral parts of male and female flowers of Laurus nobilis. *J. Agric. Food Chem.* 50, 4647–4652. 10.1021/jf020269x

# 13. Baser, K. H. C., Özek, T., Demirci, B., and Duman, H. (2000). Composition of the essential oil of Glaucosciadium cordifolium (Boiss.) Burtt et Davis from Turkey. *Flavour Fragr. J.* 15, 45–46. 10.1002/ffj.867

# 14. Cavalli, J. F., Tomi, F., Bernardini, A. F., and Casanova, J. (2006). Chemical variability of the essential oil of Helichrysum faradifani Sc. Ell. from Madagascar. *Flavour Fragr. J*. 21, 111–114. doi: 10.1002/ffj.1531

# 15. Molleken, U., Sinnwell, V., and Kubeczka, K. H. (1998). The essential oil composition of fruits from Smyrnium perfoliatum. *Phytochemistry* 47, 1079–1083. 10.1016/S0031-9422(98)80076-9

# 16. Dib, M. E. L. A., Djabou, N., Desjobert, L. M., Allali, H., Tabti, B., Muselli, A., and Costa, J. (2010). Characterization of volatile compounds of Daucus crinitus Desf. headspace solid-phase microextraction as alternative technique to hydrodistillation. *Chem. Cent. J.* 4, 16, 1-15.

# 17. Piveteau, F., Le Guen, S., Gandemer, G., Baud, J. P., and Demaimay, M. (2000). Aroma of fresh oysters Crassostrea gigas: composition and aroma notes. *J. Agric. Food Chem.* 48, 4851–4857. 10.1021/jf991394k

# 18. Muselli, A., Pau, M., Desjobert, J. M., Foddai, M., Usai, M., and Costa, J. (2009). Volatile constituents of Achillea ligustica All. by HS-SPME/GC/GC-MS. *Chromatographia* 69, 575–585. 10.1365/s10337-008-0939-1

# 19. Ngassoum, M. B., Yonkeu, S., Jirovetz, L., Buchbauer, G., Schmaus, G., and Hammerschmidt, F. J. H. (1999). Chemical composition of essential oils of Lantana camara leaves and flowers from Cameroon and Madagascar. *Flavour Fragr. J.* 14, 245–250. 10.1002/ffj.819

# 20. Bertoli, A., Pistelli, L., Morelli, I., Fraternale, D., Giamperi, L., and Ricci, D. (2004). Volatile constituents of different parts (roots, stems and leaves) of Smyrnium olusatrum L. *Flavour Fragr. J.* 19, 522–525. 10.1002/ffj.1382

# 21. Wei, A., and Shibamoto, T. (2007). Antioxidant activities and volatile constituents of various essential oils. *J. Agric. Food Chem.* 55, 1737–1742. 10.1021/jf062959x

# 22. Shimizu, Y., Imayoshi, Y., Kato, M., Maeda, K., Iwabuchi, H., and Shimomura, K. (2009). Volatiles from leaves of field-grown plants and shoot cultures of Gynura bicolor DC. *Flavour Fragr. J*. 24, 251–258. 10.1002/ffj.1938

# 23. Piskorski, R., Hanus, R., Vasickova, S., Cvacka, J., Sobotnik, J., Svatos, A., and Valterova, I. (2007). Nitroalkenes and sesquiterpene hydrocarbons from the frontal gland of three Prorhinotermes termite species. *J. Chem. Ecol.* 33, 1787–1794. 10.1007/s10886-007-9341-y

# 24. Benites, J., Moiteiro, C., Miguel, G., Rojo, L., Lopez, J., Venancio, F., Ramalho, L., Feio, S., Dandlen, S., Casanova, H., and Torres, I. (2009). Composition and biological activity of the essential oil of Peruvian Lantana camara. *J. Chil. Chem. Soc.* 54, 379–384. 10.4067/S0717-97072009000400012

# 25. Ito, Y., Sugimoto, A., Kakuda, T., and Kubota, K. (2002). Identification of potent odorants in Chinese jasmine green tea scented with flowers of Jasminum sambac. *J. Agric. Food Chem.* 50, 4878–4884. 10.1021/jf020282h
