# Supplementary material for: Scents on the slope: declining volatile emissions but stable predator-associated cues across elevation in alpine willows
Source: Front Plant Sci. 2026 Jul 29;17:1873253. doi: 10.3389/fpls.2026.1873253 (PMC13462214; doi:10.3389/fpls.2026.1873253)
Supplement: Supplementary file 4 [file Table4.docx]

Supplementary Tables

**Scents on the slope: Declining volatile emissions but stable predator-associated cues across elevation in alpine willows**

Table S4. Post-hoc pairwise comparisons for significant Generalised Linear Mixed-Effect Models (GLMMs) testing the effects of species, elevation, and plasticine caterpillar colour on bird and invertebrate predation patterns in two willow species pairs (*Salix appendiculata*–*caprea* and *S. mielichhoferi*–*myrsinifolia*).

| Contrast | Estimate | SE | Lower CI | Upper CI |
| --- | --- | --- | --- | --- |
| *Salix appendiculata-caprea* pair | | | | |
| APP Bird – CAP Bird | -1.2566 | 0.4119 | -2.3147 | -0.19847 |
| APP Bird – APP Invertebrate | -0.6681 | 0.2833 | -1.396 | 0.05986 |
| APP Bird – CAP Invertebrate | -1.0475 | 0.4019 | -2.08 | -0.01489 |
| CAP Bird – APP Invertebrate | 0.5885 | 0.4129 | -0.4721 | 1.64916 |
| CAP Bird – CAP Invertebrate | 0.2091 | 0.4579 | -0.9673 | 1.38556 |
| APP Invertebrate – CAP Invertebrate | -0.3794 | 0.4031 | -1.415 | 0.65621 |
| *Salix mielichhoferi-myrsinifolia* pair | | | | |
| contrast | Estimate | SE | Lower CI | Upper CI |
| Yellow Bird - Brown Bird | -0.178951817 | 0.423320143 | -1.385289532 | 1.027385897 |
| Yellow Bird - Green Bird | -0.619296927 | 0.423949733 | -1.827428789 | 0.588834934 |
| Yellow Bird - Yellow Invertebrate | 2.59E-06 | 0.425312954 | -1.212014047 | 1.212019235 |
| Yellow Bird - Brown Invertebrate | -1.819331595 | 0.468531921 | -3.15450956 | -0.48415363 |
| Yellow Bird - Green Invertebrate | -1.94763677 | 0.477858428 | -3.309392535 | -0.585881006 |
| Brown Bird - Green Bird | -0.44034511 | 0.421341958 | -1.64104558 | 0.76035536 |
| Brown Bird - Yellow Invertebrate | 0.178954411 | 0.423320183 | -1.02738342 | 1.385292242 |
| Brown Bird - Brown Invertebrate | -1.640379778 | 0.465432679 | -2.966725818 | -0.314033738 |
| Brown Bird - Green Invertebrate | -1.768684953 | 0.47476832 | -3.121634818 | -0.415735089 |
| Green Bird - Yellow Invertebrate | 0.619299521 | 0.42394978 | -0.588832474 | 1.827431516 |
| Green Bird - Brown Invertebrate | -1.200034668 | 0.463004621 | -2.519461456 | 0.11939212 |
| Green Bird - Green Invertebrate | -1.328339843 | 0.472264524 | -2.674154628 | 0.017474942 |
| Yellow Invertebrate - Brown Invertebrate | -1.819334189 | 0.468531976 | -3.154512311 | -0.484156067 |
| Yellow Invertebrate - Green Invertebrate | -1.947639364 | 0.477858483 | -3.309395284 | -0.585883445 |

Table S5. Partial Mantel tests assessing correlations between similarity in volatile organic compound (VOC) emissions and predation patterns (bird and invertebrate) across induction treatments in two willow species pairs (*Salix appendiculata*–*caprea* and *S. mielichhoferi*–*myrsinifolia*) while considering the effect of elevation.

| Predation type | VOC matrix | r | p |  |
| --- | --- | --- | --- | --- |
| *Salix appendiculata-caprea* pair | | | |  |
| Total bird predation | VOC abundance | 0.027475 | 0.290 |  |
| Total invertebrate predation | VOC abundance | 0.097105 | 0.041 |  |
| Total bird predation | VOC richness | -0.004786 | 0.483 |  |
| Total invertebrate predation | VOC richness | 0.037735 | 0.238 |  |
| *Salix mielichhoferi-myrsinifolia* pair | | | | |
| Total bird predation | VOC abundance | -0.03551 | 0.656 |  |
| Total invertebrate predation | VOC abundance | 0.06131 | 0.122 |  |
| Total bird predation | VOC richness | 0.04311 | 0.290 |  |
| Total invertebrate predation | VOC richness | 0.13764 | 0.020 |  |
| Invertebrate predation brown | VOC richness | 0.21071 | 0.01 |  |
| Invertebrate predation green | VOC abundance | 0.08949 | 0.048 |  |

Table S6. Generalised Linear Mixed-Effect Models (GLMMs) testing the effects of individual volatile organic compounds (VOCs) on bird and invertebrate predation patterns in two willow species pairs (*Salix appendiculata*–*caprea* and *S. mielichhoferi*–*myrsinifolia*).

| Bird predation | | | | | | | |
| --- | --- | --- | --- | --- | --- | --- | --- |
| VOC | AIC (null) | AIC (VOC) | X^2^ | DF | p | Adj-p | AIC (Δ) |
| *Salix appendiculata-caprea* pair | | | | | | | |
| β-bourbonene | -51.86 | -57.68 | 7.818 | 4 | 0.005 | 0.007 | 5.818 |
| *Salix mielichhoferi-myrsinifolia* pair | | | | | | | |
| (*Z*)-3-Hexen-1-ol acetate | -68.30 | -74.19 | 7.891 | 4 | 0.0049 | 0.007 | 5.891 |
| α-muurolene | -68.30 | -70.86 | 4.559 | 4 | 0.032 | 0.032 | 2.559 |

Table S7. Generalised Additive Models (GAM) assessing elevational trends of individual volatile organic compounds (VOCs) significantly correlated with predation rates in two willow species pairs (*Salix appendiculata*–*caprea* and *S. mielichhoferi*–*myrsinifolia*).

| VOC | GAM trend | EDF | EDF (ref) | X^2^ | p | p (adj) |
| --- | --- | --- | --- | --- | --- | --- |
| *Salix appendiculata-caprea* pair | | | | | | |
| β-bourbonene | Decreasing | 1 | 1 | 2.64725 | 0.11042 | 0.1656 |
| *Salix mielichhoferi-myrsinifolia* pair | | | | | | |
| (*Z*)-3-Hexen-1-ol acetate | Decreasing | 1.595 | 1.836 | 2.43961 | 0.06716 | 0.1343 |
| α-muurolene | Increasing | 1 | 1 | 0.03907 | 0.84416 | 0.8442 |
